# A Study of the Coevolution of Digital Organisms with an Evolutionary Cellular Automaton

**DOI:** 10.3390/biology10111147

**Published:** 2021-11-07

**Authors:** Javier Falgueras-Cano, Juan-Antonio Falgueras-Cano, Andrés Moya

**Affiliations:** 1 Institute for Integrative Systems Biology (I2SysBio), University of Valencia and CSIC, 46980 Valencia, Spain; 2 Department of Languages and Computer Science, University of Málaga, 29017 Málaga, Spain; juanfc@uma.es; 3 Genomics and Health Area, Foundation for the Promotion of Sanitary and Biomedical Research (FISABIO), 46020 Valencia, Spain; 4 Biomedical Research Centre Network of Epidemiology and Public Health (CIBEResp), 28029 Madrid, Spain

**Keywords:** evolutionary altruism, cross-species cooperation, sex ratio, phenotypic plasticity, symbiosis

## Abstract

**Simple Summary:**

We present a program that simulates the coevolution of digital organisms competing for limited resources. The rules of competition and collaboration implicit in the code have been inspired by the underlying mechanisms that generate natural selection. The simplest, most general, and least controversial rules presented in natural systems have been chosen, and these rules have been implemented in the most flexible and adaptable way found. Our model is capable of (i) measuring the evolutionarily stable equilibrium or the unstable imbalance of biological interactions, (ii) weighing biological competition and cooperation relationships to evaluate their relative incidence in the coevolution of species, (iii) verifying widely contrasted evolutionary theories with this instrument, and (iv) shedding light on the evolutionary effects of other mechanisms, such as the spatial distribution of populations, that have not been studied in depth before.

**Abstract:**

This paper presents an Evolutionary Cellular Automaton (ECA) that simulates the evolutionary dynamics of biological interactions by manipulating strategies of dispersion and associations between digital organisms. The parameterization of the different types of interaction and distribution strategies using configuration files generates easily interpretable results. In that respect, ECA is an effective instrument for measuring the effects of relative adaptive advantages and a good resource for studying natural selection. Although ECA works effectively in obtaining the expected results from most well-known biological interactions, some unexpected effects were observed. For example, organisms uniformly distributed in fragmented habitats do not favor eusociality, and mutualism evolved from parasitism simply by varying phenotypic flexibility. Finally, we have verified that natural selection represents a cost for the emergence of sex by destabilizing the stable evolutionary strategy of the 1:1 sex ratio after generating randomly different distributions in each generation.

## 1. Introduction

Coevolution refers to the reciprocal evolutionary changes between interacting organisms of the same or different species supported by natural selection [1,2]. In inter-specific coevolution, the pressure of two or more species towards mutual and synchronic selection on a geologic time scale, results in reciprocal adaptations [3]. Examples of interspecific interactions include, among others, host–pathogen, symbiosis, pollination, competition, mimesis, and predator–prey. Notably, such interactions are typically associated with another complex species network that influences the coevolutionary interactions. In the study of this so-called diffuse coevolution, the effect of abiotic factors such as weather, humidity, or nutrients is often neglected [4].

How coevolution contributes to speciation and species radiation has been a recurrent study for some time, but little progress has been made [3,5]. This is due to the inherent complexity of biological systems [6,7] and our limited ability to obtain an adequate measure of the cost of antagonistic coevolution [8,9], the degree of parenthood, or the genotypic composition of the coevolving species [10]. For instance, in the case of host–pathogen coevolution, demonstrating the effect of adaptations not only requires a long-term record [4,11], but we also need to consider that it takes place within a more extensive ecological network of additional interacting species, which can influence the coevolutionary arms race between host and pathogen [11,12,13].

Previous laboratory and field studies also present limitations when addressing the causes of coevolution. For instance, experimental evolution of short-lifecycle interacting species [14,15] are not designed to detect the effects of abiotic factors or diffuse coevolution on the interacting species [16]. In the case of field studies, the causal relationship between selective pressure and coevolutive adaptation is difficult to determine by comparative genomics between living species with a common close ancestor that has evolved in different habitats [17] or by fossil-based studies [18]. Finally, coevolutionary modeling based on integrodifferential equations [19,20,21,22], albeit general, also has a limited capacity to account for genuine cases of coevolution.

To avoid some of the limitations of these cited studies, some researchers propose the use of cellular automata [23] or evolutionary algorithms based on artificial intelligence [24]. Some of them are even used to find ingenious solutions to specific problems [25]. Simulations generally use simplified models that necessarily lack realism but test specific hypotheses under controlled conditions only. Although simulations are considered insufficient to validate a particular hypothesis or theory, they can provide good support for them [26]. Here, we present an Evolutionary Cellular Automaton (ECA) to study coevolution between species in habitats determined by initial parameters that reflect the most relevant characteristics of the corresponding populations. One of these parameters is the degree of phenotypic plasticity [27], which can generate variable polymorphism in populations even of the same genotype [28]. We implement this specific parameter that could affect all the organisms in the population (value 1), none of them (value 0), or a given proportion (value between 0 and 1) [29]. Other parameters in ECA with gradual values are intra- and inter-specific cooperation, the amount and diversity of competitive species, habitat fragmentation, and population distribution strategies of habitat cells. 

ECA compares the different predictions arising from the various theories related to the emergence of evolutionary altruism [30], to hierarchical speciation [31], and particularly to our Weighted Fitness Theory [32]. ECA is easily scalable from trivial and simplistic systems to complex associations between several species, as in the case of multilevel selection [33]. In this case, the variation in the proportion of individual and group intensity allows for the observation of coevolution of the different digital organisms with variable Hamiltonian parameters of both direct and indirect fitness [34].

It is difficult to compare ECA with similar research platforms because it is unique within the current scientific software ecosystem, although it does share certain similarities with some existing models. For example, individual-based models (*IBMs*), which are more flexible for individual action than the traditional compartment modeling approach [35], are based on three key factors: (i) the inclusion of individual variations that incorporate details about life history and age classes, (ii) the possibility for agents to adapt and learn from experience, and (iii) the modification of the environment by the behavior of the individual. ECA simplifies the underlying selective processes by avoiding these three characteristics of *IBMs*, and thus focuses the analysis on the adaptive and evolutionary balances of biological interactions, which constitutes the main objective of this work. Other models have added features such as multiple types of *CPUs* to form the bodies of digital organisms, as in some of the *EcoSim* [36,37], *Avida* [38,39,40], or *COMETS* [41] models. However, ECA uses the average of the individual traits of a species as a definition of a population of digital organisms. In addition, homogeneity and uniformity within species is a common feature of ECA. Moreover, ECA only implements mutations and phenotypic or genotypic variability gradually and as an option.

## 2. Materials and Methods

### 2.1. General Description of the Cellular Automaton

We have named this application Evolutionary Cellular Automaton (ECA) because it shares characteristics with cellular automata, specifically Von Neumann linear, finite, probabilistic, non-uniform, and non-standard automata [23]. ECA also shares aspects of Evolutionary Algorithms because it follows Evolutionary Biology principles; however, unlike these, in ECA, there is no recombination or crossing between organisms. Nor does ECA resort to Artificial Intelligence; rather, it is a dynamic system that evolves autonomously in discrete steps in a reticular network of interconnected patches [42]. In every cell in the reticular network, digital organisms concur and compete for the limited resources of the cell. Only those able to access those resources reproduce and, by natural selection, individuals compete for the limited resources in a habitat patch. A digital species represents the population or community of a real one. We can introduce the random variability of offspring fitness with different model parameters, reflecting the variations in subsequent generations of biological species. Moreover, it is a linear and probabilistic model built over an ordered group of cells in a unidimensional and dynamic landscape, which changes over generations according to a transition function, which includes stochastic values. ECA can also be considered a zero-sum game and of zero players, which means that its evolution depends on the initial stage, without the need for later data entries. ECA can model fundamentally complex interactions from a combined sum of several simple mechanisms embedded in the ECA. Finally, ECA simulates the evolution of a non-specific ecological habitat. Each simulation defines an initial stage characterized by the size and richness of the habitat harboring several species described by numerous parameters. ECA simulates interaction mechanisms among species through parameters that can enact or modulate the different interactions. 

### 2.2. Notation of ECA

The parameters defined at the initial stage are:

1 Habitat related parameters defining selective pressure:
  (a) *NumberOfCells*: number of cells that form the habitat.
  (b) *NumberOfRsrcsInEachCell*: discrete number of resources for each cell.
  (c) *Distribution*: redistribution of the populations or permeability among cells after every generation. It defines the redistribution strategy and the distance from the average after every generation.

Habitat parameters control the growth speed of the population of digital organisms. According to the Law of Constant Final Yield [43], they are a determinant factor of the population limit of organisms. It is the case of the carrying capacity [44], which imposes selective pressure among individuals (see 1LCFY.json in Supplementary Materials).

2 Species parameters that define each digital organism:
  (d) *id*: name of the species.
  (e) *NumberOfItems*: the size of the initial population.
  (f) *DirectOffspring*: number of direct offspring for each generation.
  (g) *IndirectOffspring*: number of indirect offspring or offspring given to their associates in each generation.
  (h) *Distribution*: akin to the above and defined with the habitat parameters, but each species is specified here. If it is not, the global value of *Distribution* is taken by default. This way, whereas habitat structural dispersion would be its *permeability*, species functional dispersion would be its *vagility* (i.e., the ability of a species to move about freely).
  (i) *GroupPartner*: list of identifiers (*id*) of the species with which a given species can group. Organisms in a cell are grouped with the organisms listed in their *GroupPartner* in the proportion indicated by their *PhenotypicFlexibility* parameter. To have partners requires defining not only the grouped species but also each group as a new species. Such groups are identified syntactically by joining the identifiers of each component with a vertical bar (|), e.g., A|B. Each group can be composed of two or more partners, e.g., A|B|C.
  (j) *PhenotypicFlexibility*: as explained in the definition of *GroupPartner*, it is the proportion of organisms of the species that must be grouped. When applied to the group, *PhenotypicFlexibility* defines the proportion of that group that remains grouped into the next generation—the greater the *PhenotypicFlexibility*, the more significant the number of groupings in the habitat.
  (k) *AssociatedSpecies*: *id* list of identifiers with which a given species is associated. As we will see below, one of the differences between grouping and association-based interactions is that all the organisms that meet their associate are associated with the latter. In contrast, grouping is only possible according to the proportion indicated in their *PhenotypicFlexibility*, as previously indicated. It is also remarkable that, whereas groups are new organisms with characteristics that are different from those of their components, associations are dynamic forms that do not generate new organisms but only indicate cooperation between species.
  (l) *FitnessVariationLimit*: maximum variation of *DirectOffspring* in the event that random variations of *DirectFitness* and *IndirectFitness* parameters are allowed. In any case, the sum of both is always constant.

### 2.3. Fitness of the Model

We test ECA fitness with a training dataset obtained from fieldwork, lab experiments, or data freely introduced by the user (see Supplementary Materials, reference column). Such a dataset allows for the configuration in different simulations of species parameters that can reflect biological interactions, i.e., mutual benefit, selfishness, altruism or resentment [45], predation [46], amensalism, parasitism [47,48], exclusion [49], intraspecific competition, neutralism [50], commensalism [51], protocooperation [52], intraspecific social cooperation, subsociality, or symbiosis (see Table 1).

### 2.4. Characteristics of ECA

Most of the characteristics that influence the evolution of populations are considered to be fixed in ECA, which means that they do not vary between generations, species, and cells. They are necessary factors for natural selection, so they are fixed in ECA for simplicity. The main fixed factors are:

(a) *Immutable biological efficiency*: a consumed resource unit always provides a descendant.
(b) *Resource uniformity*: every cell has the same resources. Every cell representing the dynamic patches that divide the habitat [53] has the same amount of resources at the beginning of every generation, and the extra resources of each generation disappear for the next.
(c) *One-to-one interactions*: in every cell, each simple or grouped organism relates to each other by pairs, 1 to 1. If an organism interacts with a list of species, the number of couples with each species is set up proportionally to their populations, as we assume that the greater the population density, the higher the probability of interaction.
(d) *Randomness accessing resources*: there are no hierarchies to access resources; every organism has the same opportunities to obtain them by queuing randomly.
(e) *Simultaneous generational changes*: generational changes are simultaneous for the whole habitat so that all the organisms consume their resources and have their offspring simultaneously.
(f) *Parents do not survive replication*: only descendants survive the next generation.
(g) *There are no mutations*: mutations are not considered in studying the effects of the parameters that are the objective of our study. Nevertheless, ECA can introduce mutations in species by (i) pausing the execution of the simulation; (ii) modifying the values to mutate in the intermediate configuration automatically saved in the folder cont.json; and (iii) by restarting the execution.
(h) *Habitat changes are not considered*: for the same reasons as above, habitat changes can be introduced manually following the above-mentioned instructions.
(i) *Limited variability*: we have implemented the argument *varia* to study the influence of selective pressure on species association capacity through variability and phenotypic accommodation of the associations [54]. The *varia* parameter sets random variability in the *DirectOffspring* of the descendants. The range of such variability is set with the parameter *FitnessVariationLimit*, which is also configured initially. Nevertheless, as nature limits evolutionary adaptations, the sum *DirectOffspring* + *IndirectOffspring* = *InclusiveFitness* of each organism will always remain unchanged, avoiding the coevolutionary “arms race” in biological fitness [55].

### 2.5. Other Features Not Considered in ECA

ECA is unable to implement other characteristics that are not generalized traits, even though they may be significant in the evolution of particular species, such as sex change in some fish species [56]. ECA abstracts the non-general characteristics, focusing on several parameters to quickly calculate the most influential factors in species coevolution: fitness and selective pressure.

### 2.6. ECA Is Multi-Hierarchical

A digital organism in a patch can represent any replicator that consumes limited resources and is situated at any level of the biological hierarchy, such as an allele, a gene, a group of genes, an individual, a sexual partner, or a biological population or subpopulation. The digital organism represents the interactor upon which natural selection acts [57], the selection unit that competes against other organisms of the same species or other species. ECA can model any level of biological hierarchy [58], the digital organism under study can range from genetic entities to complex organisms, either individual or group formation. In ECA, different digital organisms concur, as communities of different species cohabitate in each real patch. The state of a patch depends on the number of digital organisms of each population. Organisms that concur in a cell consume its limited resources (*NumberOfRsrcsInEachCell*) and reproduce individually or interact with others. The neighborhood of each cell is established by the surrounding ones, representing the cells in the same generation. All those surrounding and coetaneous cells constitute the whole reticular network and represent the ecologic habitat. A transition function determines the status of the habitat in the next discrete step.

### 2.7. Biological Interactions in ECA

Interaction between two organisms can occur either by association (that does not create a new organism) or by grouping (that creates one).

*Interaction by association*. Analogously with Hamilton’s concepts [34], each organism is defined by two parameters: direct offspring or direct biological fitness (*DirectOffspring*), which is the fitness of the organism given by the number of direct offspring; and indirect offspring or indirect fitness (*IndirectOffspring*), which is the effect of the digital organism (the actor) on the reproduction of another organism with which it is associated (the recipient) [45], also called neighbor-modulated fitness [10] (Table 2).

Each digital organism consumes a part of the cell’s resources to maintain its basal metabolism, reproduce, and undertake all other vital activities for each generation. That amount is its *Inclusive offspring*. The cost of association of two digital organisms (*IndirectOffspring*) increases the resources consumed. Each organism’s offspring on each discrete step would be its personal offspring. As the consummation of cell resources constitutes the biological cost of the organism, its offspring is its biological benefit (Table 3). 

**Figure 1:**
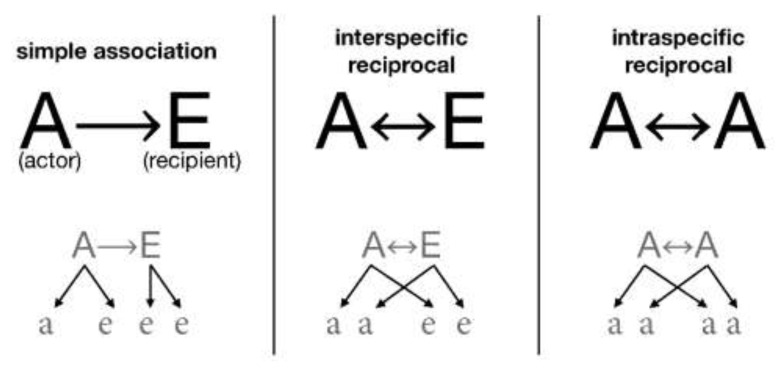
Class of associations. Chart of the type of associations among digital organisms. A and E are organisms with their offspring (a and e, respectively). The arrows indicate the direction in which the resources are delivered.

If there is no association, the digital organism consumes resources and has its offspring as *DirectOffspring*. Each organism can associate with any other as an actor, providing their resources, or as a recipient, receiving from the other’s resources. The relationship can be inter-specific reciprocal when both species give and take simultaneously; when they are of the same species, it is a case of intra-specific reciprocal. Figure 1 presents the chosen syntax under which we defined these interactions.

**Figure 2:**
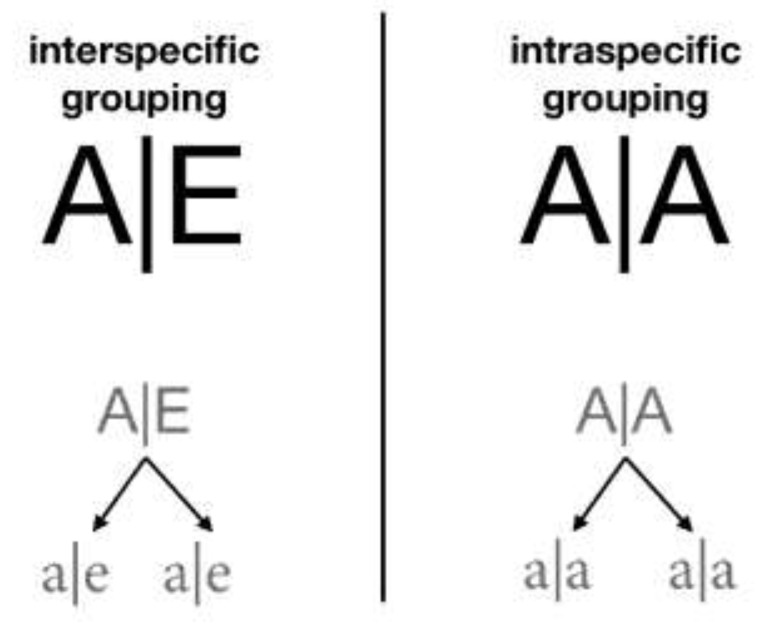
Class of groupings. Chart of the types of groups among digital organisms. A|E and A|A are group of organisms with their offspring a|e and a|a, respectively). The arrows indicate the direction in which the resources are delivered

*Interaction by grouping*. Other than by association, two digital organisms can interact by grouping. It implies a more intimate relationship, creating a more complex entity with new species characteristics, which are different from the composting organisms. Grouping represents interactions of mutualism and eusociality, depending on whether they are inter-specific or intra-specific, respectively. Groups are syntactically represented as A|B, where A and B are the *id* of the grouped species. As groups constitute new entities themselves, they can successfully group with other entities, creating multi-organism groups like A|B|C (Figure 2).

The concept of phenotypic flexibility reflects the sub-sociability relation and is defined by the probability *PhenotypicFlexibility*. Even the simplest organisms, such as bacteria, can detect an environmental change [59], which sparks a genetic trigger [60]. This favors a change in the phenotype that groups independent individuals into a multicellular structure, a mass of individuals, or fructiferous bodies. Some die when the structure is formed. Others transform into spores or resistant forms that remain latent as if they were asleep until food becomes available again [61,62,63]. 

Environmental characteristics cause changes in phenotypic expression. These environmental characteristics include lack of food or absence of a partner [64]. In biology, quorum sensing is the ability to detect and respond to cell population density by gene regulation [59,65]. In ECA, we assume that digital organisms detect the number of conspecifics and will group—or not—depending on the value of their *PhenotypicFlexibility*. The larger the proportion of *PhenotypicFlexibility*, the greater the number of groupings will be. 

Bet hedging [66] in Evolutionary Biology occurs when organisms decrease their biological fitness under specific conditions in exchange for increased biological fitness in possible stressful conditions. We can approximate this strategy in ECA by keeping a proportion of the grouped organisms in the next generation. To do so, the greater the *PhenotypicFlexibility* of the group, the more numerous the grouped forms remaining in the next generation will be, which guarantees higher or lower levels of bet-hedging.

### 2.8. Transitional function in ECA

**Figure 3:**
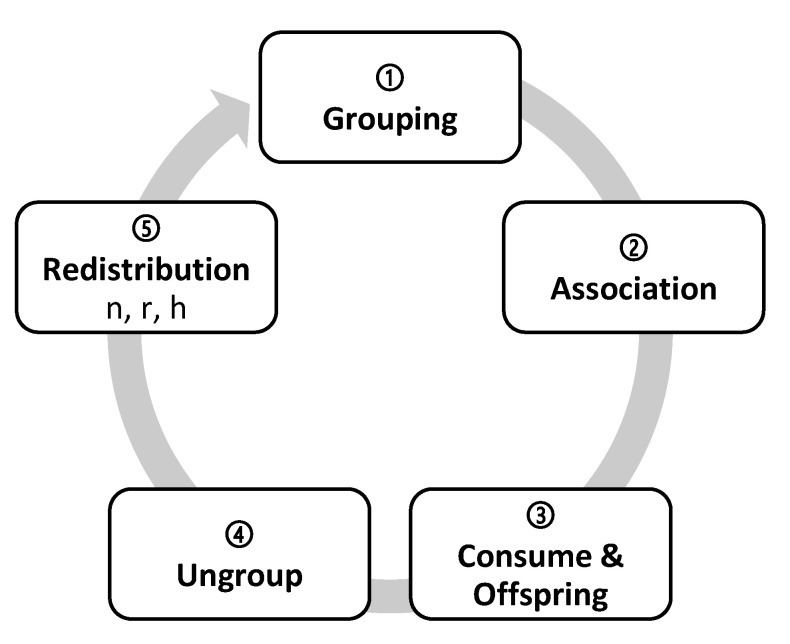
Transition function. Stages of the transition function. There is first a random distribution of organisms throughout the reticular network of cells, and later on, these stages are executed for each generation.

The transitional function in ECA determines the state of every cell for the next generation depending on its previous state and its neighborhood (see Figure 3). Just once, and before ECA iterates with this transition function, there is a random distribution of the digital organisms (*NumberOfItems*) among all the cells of the reticular network (*NumberOfCells*). The transition function repeats the following steps in each generation:

(a) Grouping (*doGrouping*): the organisms that have one *GroupPartner* or more are grouped randomly, in proportion with the *PhenotypicFlexibility* of the first grouped organism.
(b) Association (*doAssociation*): organisms with associates associate with them and can take up to five possible association roles: solitary, actors, recipients, intra-specific reciprocal, and inter-specific reciprocal (Figure 1).
(c) Consumption and replication (*doEnqueuingConsumeAndOffspring*): organisms randomly queue to eat and reproduce. As long as there are resources they simultaneously eat and have their offspring, either directly (*DirectOffspring*), that they give to their species, or indirectly (*IndirectOffspring*), that they give to their associate. With this, randomness is implemented in access to resources, as the likelihood to occupy a specific spot in the queue is the same for all the organisms. We have studied three ways for the associates to access resources: (i) to place the associates together in a single spot of the queue; (ii) to place them in two different spots so that when is the turn of the first, it calls upon the second to consume together; and (iii) as in the second option, to place them independently but when is the turn of the first, it does not call upon the second to consume together. In the first option, associations are penalized, as they have half the chance that solitary organisms do to access resources; in the second option, associations double their chances, as they take two spots in the queue and either of the organisms ensures that both consume. We finally chose the third option, in which its recipients who do not consume will not have direct offspring, but they could receive indirect offspring as a result of the actor recipient interaction.
(d) Ungrouping (*doUngroup*): recently formed groups ungroup proportionally to their phenotypic flexibility. The most complex groups must be ungrouped first, followed by the simplest ones to reach the maximum number of ungrouping. The more *PhenotypicFlexibility* the group has, the more groups remain.
(e) Redistribution (*doDistribute*): preparing the next generation of descendants, they distribute between the cells, more or less uniformly. Depending on the cells involved, the distribution can be (i) local (combining the neighbors only) or (ii) global (ignoring the place of origin). There are, therefore, two strategies: (i) The *n* strategy of *neigbours_distribution* generates a local distribution: descendants randomly distribute from one generation to the next, one by one but within a range of neighboring cells. The user can set the range of adjacent cells or neighborhood range in the initial configuration (*Distribution*) from the value *0n*, in which organisms remain in their cell of origin, to the value *100n*, in which organisms distribute among any of the cells of the reticular network. According to the law of large numbers, the distribution becomes more uniform for larger populations and broader ranges, decreasing the variance. (ii) The *r* strategy of *random_global_avg* generates a global redistribution: all the organisms globally disperse, regardless of their origin. Groups of random size are constructed and assigned to target cells randomly. Although the neighborhood range is maxed out, the whole reticular network can distribute from *0r* with zero variance (as all the values are the same) up to *100r*, where each cell receives a random number of organisms, thus obtaining a high variance. The *h* strategy of *random_global_by_cell* is another alternative: the number of target cells is reduced while the distribution *r* takes place, increasing the variance with respect to the mean. This produces a higher or lower number of empty cells depending on the chosen value of *h*. Thus, *0h* means no empty cells and variance 0, where all the values are the same (equivalent to *0r*), whereas *50h* means a distribution with 50% of empty cells and 50% of randomly occupied cells also with a distribution *50r*. The extreme value *100h* means that all organisms end in only one cell.

We use the terms *permeability* and *vagility* instead of *connectivity* to refer to the three types of distribution because Conservation Biology commonly defines connectivity as the capacity of individual species to move among habitat areas through corridors and connection areas [67]. Consequently, the analysis of landscape connectivity typically implies identifying connections between specific places regarding a particular species [68]. By contrast, our theoretical model requires a more extensive and more inclusive analysis that refers to a more general landscape, in which any real system from any biological hierarchy can be represented. We, therefore, differentiate between permeability (as structural connectivity related to the landscape) and vagility (as functional connectivity specific to each species).

For each initial configuration analyzed, ten simulations are run. If similar results are produced despite the stochastic nature of the model, any one of them is chosen as representative. However, if the results are different, as is the case with genetic drift, each possible result is presented.

### 2.9. Software

We have used free software (Python version 3.9) with numerical libraries *numpy* and graphic libraries *matplotlib* and *gnuplot*. We have employed the utilities of the operating system UNIX in OSX and Excel for intermediary tests. The program can be accessed at Zenodo (https://doi.org/10.5281/zenodo.5639551 (accessed on 2 November 2021). For additional details to run *ACE*, contact J.F.-C. (Javier Falgueras-Cano).

## 3. Results and Discussion

### 3.1. ECA as a Virtual Lab

We have simulated different evolutionary theories with ECA (Table 4). For example, the functional and numeric responses generated by the changes in predator/prey density [46] (1Pred.json in Supplementary Materials), or how amensalism would vary with or without a cost for the actor [69] (1Amen.json and 1Amen2.json in Supplementary Materials). Modifying the available resources, we can even predict in which generation the principle of Hardy Weinberg [70] will not be fulfilled (1Hardy.json in Supplementary Materials).

### 3.2. Specific Scenarios Do Not Favor Collaboration

**Figure 4:**
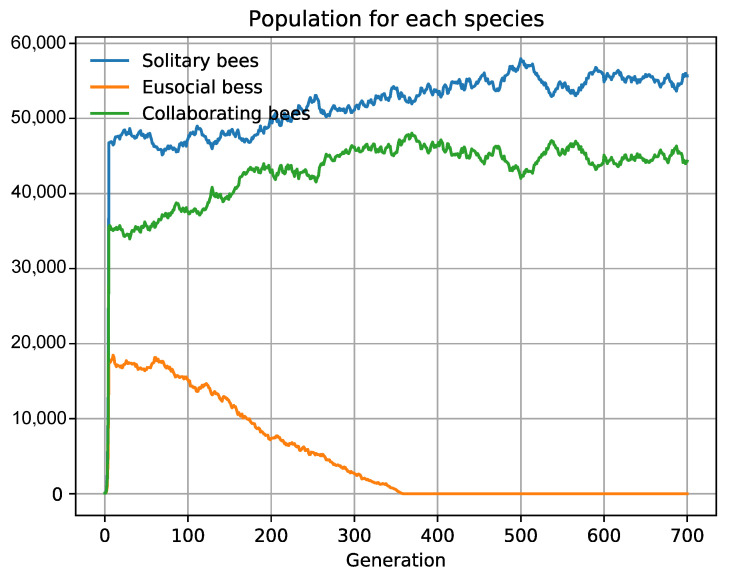
Uniform fragmentation and distribution do not favor social animals. Three types of bees compete, *ceteris paribus*. Eusocial bees are extinguished, and intra-specific collaborators have a smaller population when the habitat is fragmented (100 cells) and uniform distribution (*100n*). The need of social animals to interact thwarts their biotic potential when the habitat does not favor interaction, as occurs with uniform distribution and fragmented habitats.

We have studied the influence of habitat fragmentation and distribution type in three hypothetical bee species (1Colab.json in Supplementary Materials) [71,72]: bees with an independent life (A), bees who collaborate (B), and eusocial bees (C). The study sets the three species (with equal biological fitness and population size) in the same habitat and competition. We observe that the associations are not advantageous and that the equilibrium is maintained in all the scenarios, with no dominant species. Nevertheless, in fragmented habitats with a high number of cells and uniform distribution (e.g., 100 cells with distribution *100n*), the bee with an independent life A prevails over the other two, B and *C*. A simple explanation is that all the individuals of the independent species *A* can always have their maximum offspring. By contrast, for collaborating B and eusocial *C* bees, if the population size is odd, the uncoupled bee does not interact and only produces its *DirectOffspring*, losing the *IndirectOffspring*; if it is eusocial, it will even have no offspring. Any decrease in the interacting capacity of social organisms implies a decrease in their biotic potential. The uneven phenomenon thwarts the interaction capacity between organisms for each cell, which multiplies with the increase in habitat fragmentation (Figure 4). We also observe that the more uniform the redistribution of descendants, the more populations profit from the resources, and more organisms survive, making species fitter to compete by natural selection.

**Figure 5:**
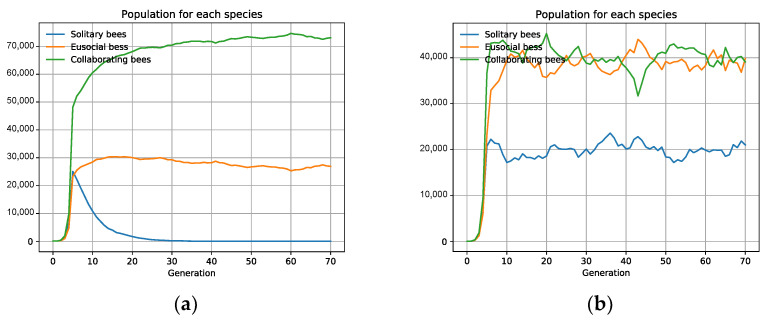
If social bees have greater fitness, they prevail over solitary ones, but only in uniform distributions (**a**). In both charts, solitary bees are less efficient with a *DirectOffpring* of 4 instead of 5. However, they only survive if there is an aggregated distribution of *100r* (**b**).

If we keep the uniform distribution *100n*, solitary bees *A* are only extinguished when we diminish their biological fitness. If we, for example, decrease the *DirectOffpring* value from 5 to 4 only for the solitary bees A, the species disappears, with the collaboration of B and *C* prevailing over A’s selfishness (Figure 5a). Nonetheless, an aggregated distribution (e.g., *100r*) can make the solitary species *A* survive with even less biological fitness than the others (Figure 5b).

**Figure 6:**
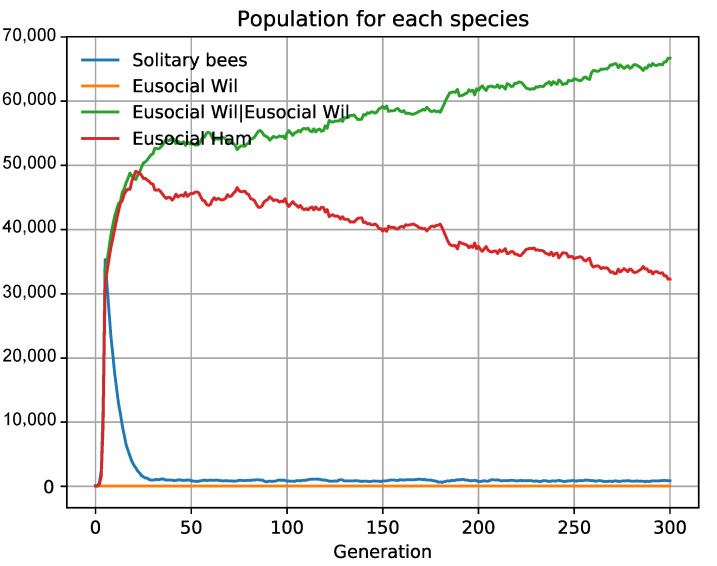
The theories of multilevel selection and kin selection are functionally equivalent. To represent eusociality, we use multilevel selection or kin selection in two different species. Neither prevails, the two species remain in evolutionary equilibrium. This means that both theories explain the same concept and are equivalent in their operation.

Hamilton’s theory perfectly explains the emergence of eusociality due to higher biological fitness, which appears under the conditions mentioned above [73]. The multilevel selection theory can also explain it. This theory conveys that the organisms of the same species can unite and cooperate to improve their biological fitness. The experiments wherein two sweat bees of the same species were forced to be together, exemplify these groupings. Such experiments were run with different sweat bees of the genus *Ceratina* and *Lasioglossum* [64]. The bees divided their work into several activities: the building of the nest, food search, and surveillance, presenting a natural predisposition, like a genetic trigger, towards self-organization. This relates to the model of a “fixed threshold” for regulating division of labor in eusocial species [73]. The fixed threshold model conveys that organisms are susceptible to changing activity when they detect certain types of variation in the environment. Such adaptation occurs by a simple change in the nervous system—replacing a certain allele—which produces phenotypic plasticity. Given that they are alternative phenotypes of the same genotype, these sub-social animals can cross the threshold to eusociality. They can also work back to solitary life if they merely spot a colleague working on an activity. We have implemented such a phenomenon in ECA by grouping two bees under the *id* Wilson eusocial, creating the new organism *Eusocial Wil|Eusocial Wil*. One is the queen and the other one the worker (see 1Eu2.json in Supplementary Materials). We made them compete simultaneously against eusocial bees associated under the kin selection theory (*id Eusocial Ham*), and against other solitary bees (*id Solitary bees*). As a result, both eusocial bees prevail over the solitary ones if the collaboration improves their biological fitness (in our case, one more descendant). Given that both species follow eusocial theories and remain in adaptive equilibrium, we can confirm that both theories are functionally equivalent (Figure 6) [74,75]. This requires maximum phenotypic flexibility of the main group (*PhenotypicFlexibility* = 1), that is, phenotypic plasticity must exist to allow the maximum number of groupings without diminishing its biotic potential.

### 3.3. The Emergence of Obligate Mutualism

The study of mutualism in populations of the stinkbug *Plautia stali* [17] demonstrates that it is possible to evolve from parasitism to endosymbiosis by natural selection. In the aforementioned study, they report the interactions between *P. stali* and a lineage of six endosymbiotic bacteria distributed among different Japanese islands [17]. Our model explains the five scenarios where the transition from a free life to obligate endosymbiosis is detected (see 1Symb.json in Supplementary Materials) by varying the phenotypic flexibility (*PhenotypicFlexibility*). Interestingly, stinkbugs were responsible for this evolution as they used bacteria to obtain their nutrients [17]. Our simulations suggest that the increase in fitness of the vertical transmission by oviposition is enough to cause this phenomenon. Stinkbug’s parents pollute the surface of their eggs with bacteria to transfer them to their offspring. As indicated, With ECA we can simulate the increase in fitness of the observed transmission mechanism by increasing the *PhenotypicFlexibility* of the so-called stinkbug|bacterium group, so that a significant proportion of bacteria become endosymbiotic. This diminishes the population of free-living bacteria, even up to their extinction, with symbiosis prevailing. To verify this experimentally, it would be enough to count the bacteria that become endosymbionts in the islands of southwestern Japan, where obligate endosymbionts and parasitized bacteria coexist, and compare this number with that of the rest of the islands.

### 3.4. The Well-Known Random Genetic Drift as a Selective Phenomenon in ACE

To consider the genetic level in the biological hierarchy, we can simulate that each allele is a digital organism, and each habitat cell is a locus. Each cell needs a minimum of four resources (*NumberOfRsrcsInEachCell* = 4) to limit the number of alleles per locus to two and allow them to replicate two by allele (*DirectOffspring* = 2). This guarantees the three possible scenarios in the future dynamics of each cell: from [A,B] you can obtain [A,A], [B,B], and [B,A] or [A,B]. In this context, random genetic drift is the only reason why an allele would prevail over another. These simulations have allowed us to estimate the number of loci needed to minimize the effect of random genetic drift [76,77] (see *1Deri.json* in Supplementary Materials). A group of cells under 100 generally demonstrates a strong effect of random genetic drift, whereas a group over 1000 cells strongly minimizes that effect. As a stochastic phenomenon that creates random genetic drift, we found that sampling errors influence the higher hierarchical levels equally. Populations can be randomly extinguished at the individual level, even without the intervention of natural selection [78]. Sampling errors extinguish some species in initial configurations without adaptive differences (see 1DeriSP.json in Supplementary Materials). ECA provides a minimum population from which there cannot be random genetic drift.

### 3.5. Kin Selection and Relevance of Benefit in Fitness

ECA can simulate the cooperation between species so that such cooperation is profitable, having more offspring, and following Hamilton’s rule *rB* > *C* (the benefits of cooperation by the relatedness ratio must outweigh the cost of such cooperation, where *r* is the coefficient of relatedness, and *B* and *C* are benefit and cost, respectively). We have implemented several empirical studies that support Kin selection [79]. We wonder, for instance, why some wild male turkeys partner to compete against other solitary males if the subordinate male of the coalition does not have offspring [80]. Although the partnered turkeys are related, and thus fulfill Hamilton’s rule, under the principle of competitive exclusion, even if the benefit is small (we only added one extra kin for the coalition), in a few generations solitary turkeys will be extinguished (see simulation 1 in 1Pavo3.json in Supplementary Materials). Unless there is a control mechanism like the green beard effect under which partnered turkeys locate and recognize each other, partnered turkeys will also be extinguished for the same reason (see simulation 2 in 1Pavo2.json in Supplementary Materials). In any event, coevolution of wild turkeys that partner and other solitary ones cannot remain in equilibrium.

**Figure 7:**
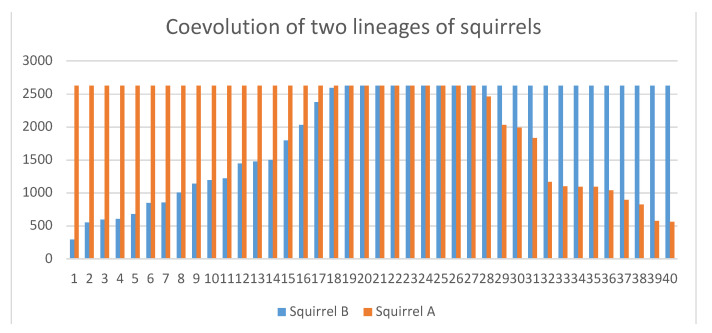
Coevolution of two lineages of squirrels (data obtained from 1Squi.json in Supplementary Materials). The x-axis represents the 40 simulations arranged in increasing order, and the y-axis the organisms. After 40 simulations, the adopting squirrels (orange) prevail over those who do not adopt (blue). If there were no adaptation advantages, the population would vary by random genetic drift, and the chart would be more symmetrical.

The red squirrel *Tamiasciurus hudsonicus* presents another interesting case, as it adopts the children of its kin neighbors. Why would adoptive parents incur the cost of raising additional distant kin? We have introduced a factor in our model (*Q* factor) that appears as often as it does in the reference study [81] and provides extra kin for the lineage A to the squirrels who adopt, while not affecting the other competing lineage B. Fieldwork provides statistical data that enabled us to configure an initial stage with relative, synchronized, and average values. We ran 40 simulations, sufficient to observe the evolutionary tendency (see 1Squi.json in Supplementary Materials). Thus, in 18 simulations, B was extinguished, 13 *A* was extinguished, and 9 remained stable after 2631 generations, equating to 10,000 years (considering a generation time of 3.8 years). Without factor *Q* or the benefit of adoption, both lineages would only be affected by ecological drift [78] at 50% (1DeriSP.json in Supplementary Materials shows the ecological drift phenomenon in a general case). However, with factor *Q*, lineage A of the adopting squirrels prevails slightly more than 16% over lineage B (Figure 7). It is questionable whether such adaptation is sufficiently strong.

Even though Hamilton’s inequality is fulfilled in both studies, we see that in one of them, adaptation can be strong enough to make coevolution in equilibrium impossible, due to the quick extinction of one of the lineages; and in the other, adaptation may be so weak that it barely outweighs the effects of drift. This increases uncertainty over the results of the reference studies of squirrels and turkeys. Therefore, the adaptive importance of a benefit must be calculated on a case-by-case basis to really analyze whether it is significant to the evolution of a population. ECA is a helpful tool in this regard.

### 3.6. The Evolution of Sex

Sex appeared very early on in the history of life on Earth [82] despite the high cost for the common ancestor of all sexual animals, namely, a 50% reduction in the chances of leaving copies of their genes, the so-called “the twofold cost of sex” [83,84]. The adaptive benefits for a species to become sexual need to outweigh its costs. Such advantages could be related to mutations or a quicker adaptation to the environment in groups and individuals [85,86,87]. The Red Queen hypothesis argues that, due to their higher genetic variability and flexibility to adapt, sexual species outdo asexual ones in stable environments with high density and high rates of parasites and pathogens that exploit the host population [88,89]. We studied this in ECA by simulating competition between two species equal in biological fitness and other initial parameters: a sexual one with males and females and an asexual one. In our simulation, if males and females do not associate, they do not have offspring (*DirectOffpring* = 0), and when they do, they have eight children, four male and four female (*IndirectOffpring* = 4), whereas the asexual species has four children (*DirectOffpring* = 4). This way, we eliminate the two-fold cost of sex because the number of descendants that are like their parents is four in both species, and we obtain results that indicate whether the benefit is for the group or the individual, or if it is long or short term (see simulation 1 in 1Sex2.json in Supplementary Materials).

**Figure 8:**
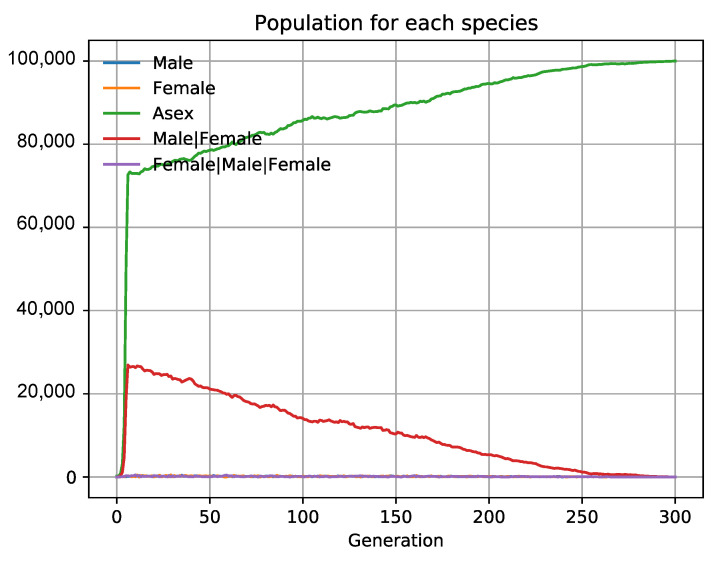
In polygamy, asexual organisms also prevail (data obtained from 1Sex3.json in Supplementary Materials). In groups that work the same way as associations, a male can partner, forming a group with only females (Male|Female) or two (Female|Male|Female). Under the same initial conditions, ceteris paribus, asexual populations prevail and extinguish sexual partners as happened in systems of monogamous associations. Only females can reproduce, and due to sampling errors, fluctuations in female populations can decrease the biotic potential of sexual populations over that of asexual ones, where they all have offspring.

We observe in the simulation that asexual species always prevail over sexual ones because each cell and generation have males or females that do not associate (singles) and do not have offspring, whereas all asexual species have offspring. This is due to the so-called *quorum decreasing* effect under which sampling errors alter the rate of male and female populations, making the number of associated couples match the lower quorum of the two associated populations. In such a monogamic system, a male needs to “associate” with a female to reproduce. However, as we know, only one male can fertilize one or many females [90]. We have configured a simulation with groups (see simulation 6 in 1Sex3.json in Supplementary Materials) to observe whether the quorum decreasing effect also influences the females of a polygamous society, thus having similar consequences to those in the monogamous species referred to before. As we have seen with eusocial bees, the groups are functionally equal to associations when the *PhenotypicFlexibility* = 1 in the main group. In these groups, a male can procreate with one or two females in only one generation. We have checked that asexual species always prevail and extinguish the sexual partners if they all have the same biological fitness (Figure 8). Sampling errors seem to be inherent to every population selection, so they are therefore general, while the other costs proposed in the literature (like finding and choosing a sexual partner) are highly variable among species and circumstances that are not considered here.

To implement the Red Queen hypothesis, we added a pathogen to the simulation (its *NumberOfItems* went from 0 to 500), which infects both populations when they group with the organism (see simulation 2 in 1Sex2.json in Supplementary Materials). We controlled contagion with the *PhenotypicFlexibility* of the group and lethality with the biological fitness of the group. So, the greater the *PhenotypicFlexibility*, the higher the proportion of groups becoming infected. On the other hand, the lesser *DirectOffpring* or *IndirectOffpring* of the (infected) group, the more lethal the pathogen is. In every generation, all the groups ungroup (*PhenotypicFlexibility* = 0 of the group), which means that the descendants from the infected ones do not become infected themselves and that the pathogen is active for the next generation. We found that when the infection index and lethality are equal for both species, for values of *PhenotypicFlexibility* = 0.5, 0.95 or 1, asexual individuals prevail over sexual couples, as sampling errors continue favoring the asexual ones.

**Figure 9:**
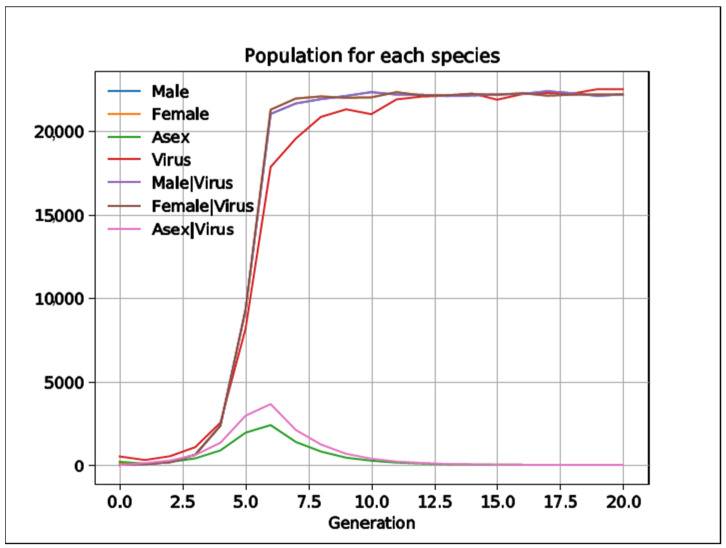
If the infection rate and lethality are higher among asexual species, sexual species prevail (data obtained from simulation 8 in 1Sex2.json in Supplementary Materials). When infections and pathogens’ lethality are simultaneously higher in the asexual populations, the sexual populations quickly overcome them.

The pathogen can become more lethal or contagious among asexual species, but not enough to extinguish them, e.g., if the infection index for asexual species is 75% and of sexual ones only 50% (changing *PhenotypicFlexibility* from 0.5 to 0.75), asexual species still prevail (see simulation 4 in 1Sex2.json in Supplementary Materials). It is noteworthy that sexual populations are superior in the first generations, while they are still infected, but extinguish later on when the epidemic is over. By contrast, asexual species also prevail if we change lethality to 1 for asexual and 2 for sexual (see simulation 5 in 1Sex2.json in Supplementary Materials). Only in the following circumstances are asexual species extinguished quickly: (i) when we make the pathogen very contagious for asexual species (from 0.5 to 0.8 for asexual *PhenotypicFlexibility*; see simulation 3 in 1Sex2.json in Supplementary Materials); (ii) when we make the pathogen very lethal when infected sexual species have three descendants while asexual have only one (see simulation 7 in 1Sex2.json in Supplementary Materials); or (iii) a mixture of both (Figure 9; see also simulation 8 in 1Sex2.json in Supplementary Materials).

Although the aforementioned study is valid for panmictic populations with one-to-one relationships, we must assume that the cost of sex is even greater in non-panmictic species where other factors can increment bias between sexes, like finding and choosing a sexual partner or sexual selection itself, increasing single populations without offspring and diminishing the biotic potential of the sexual species. Fisher’s principle sets a *sex ratio* 1:1 as an evolutionarily stable strategy that prevails over any other [91], which also agrees with the ECA results (see 1Fish.json in Supplementary Materials). Therefore, the cost of sex in the example considered in ECA is small, but it would be bigger in a natural system for the reasons explained above. The Red Queen Effect would have to compensate by providing a higher relative benefit in natural systems. The parameters that regulate lethality and degree of infection in our model (group *PhenotypicFlexibility* and *DirectOffspring*) allow us to implement any bias that would outweigh the cost of sexuality. We, therefore, highlight an unforeseen evolutive cost of sexuality: the one created by the drift effect. Such a cost is general and affects all species equally. On the other hand, the difference implied by the Red Queen Effect between sexual and asexual species has to be significant enough to make sexuality prevail in any population.

## 4. Conclusions

ECA is a valuable tool to verify evolutionary theories based on several relevant parameters. Its simplicity is the result of a long study wherein more complex prototypes were discarded. We developed the latest version using computing tools and current standards to facilitate analysis and modifications. Even if an interpreted language compromised the execution efficiency, such a choice is widely justified in this kind of modeling [92]. Still, ECA has demonstrated a reasonably efficient execution in situations involving many cells and organisms, close to similar modeling applications. ECA can be used as a virtual experimentation field in Evolutionary Biology. Furthermore, it also has other applications, i.e., a simulator of relevant parameters in evolutionary processes. Even more interestingly, it can shed light on the statistical mechanisms inherent to natural selection. Some of them, like the *quorum decreasing* effect or the importance of dispersion strategies, have not been widely researched and reveal exciting fields of study for future work.

We have proved that ECA is flexible and easily capable of recovering characteristics, traits, behaviors, qualities, and parameters of coevolutionary processes. We have chosen the suitable ones to verify universally accepted evolutionary processes. Furthermore, the model can be turned into an in silico lab to analyze more controversial processes. Nevertheless, ECA offers a very simplified version of coevolutionary processes. We have added some restrictions like offspring selection as the sole parameter to determine the biological efficiency of species, ignoring the biological efficiency of the offspring based on the resources available to them.

On the other hand, when the biological activity itself would affect the abundance of resources, selective pressure has been limited only to the number of cells and to the number of fixed resources per cell. Finally, the geometry of the represented habitats has been simplified to a linear distribution, instead of larger dimensions. All these abstractions impose limitations when applied to natural systems. Furthermore, the characteristics of some natural systems cannot be recovered directly. Thus, to simulate them, it is sometimes possible to use the complex configurations of the available parameters.

## Figures and Tables

**Table 1:** Summary of simulations to check social relationships.

Relations	File	Results in ECA
Predation	1Pred.json	Resources are prey
Amensalism	1Amen2.json	Eucalyptus kills other plants
Parasitism	1Para.json	The cuckoo survives
Exclusion	1Exclu.json	The most efficient excludes the other
Intra-specific competition	1Intra.json	Only the fittest live
Neutralism	1Neu.json	They keep in balance
Commensalism	1Comm.json	Only one benefits
Proto-Cooperation	1Proto.json	Proto-cooperation prevails
Intra-specific social collaboration	1Colab.json	Social bees prevail
Subsociality	Symb.json (simulation 4)	Sometimes solitary and other mutualists
Symbiosis	Symb.json (simulation 5)	Symbiosis prevails

See Supplementary Materials for the full list of simulations.

**Table 2:** Abbreviations of magnitudes for interactions.

Role	Direct Offspring	Indirect Offspring	Personal Offspring	Inclusive Offspring
Actor	$Df_{a}$	$If_{a}$	$Pf_{a}=$ $Df_{a}$	$F_{a}=Df_{a}+If_{a}$
Recipient	$Df_{r}$		$Pf_{r}=$$Df_{r}$ + $If_{a}$	$F_{r}=Df_{r}$
Reciprocal	$Df_{r}$	$If_{r}$	$Pf_{r}=$$Df_{r}$ + $If_{a}$	$F_{r}=Df_{r}+If_{r}$

The abbreviations and the way they were obtained are based on Hamilton’s concepts. The rows represent the roles of each digital organism, and the columns represent the offspring. For example, *Pfr* is the recipient’s offspring, similar to the Hamiltonian concept of personal fitness.

**Table 3:** Consumption and offspring of associations.

Partnerships	Individual	Recipient	Actor	Reciprocal
Consumption	$Df_{i}$	$Df_{r}$	$Pf_{a}$	$Pf_{r}$
Offspring	$Df_{i}$	$Df_{r}+If_{a}$	$Df_{a}$	$Df_{r}+If_{a}$

Calculation of consumption and offspring for organism type according to the type of association.

**Table 4:** Table summarizing simulations to verify principles and evolutionary rules.

Theories	File	Results in ECA
Law of Constant Final Yield	1LCFY.json	Resources control population
Numerical and functional answer	1Pred.json	Resources grow, the population grows
Competitive exclusion principle	1Exclu.json	The most efficient excludes the other
Random Genetic drift	1Deri.json	It tends towards homozygosity
Ecological drift	1DeriSp.json	Populations are extinct due to sampling errors
Hardy–Weinberg principle	1Hardy.json	Allele balance without natural selection
Fisher’s principle	1Fish.json	The sex ratio 1:1 prevails

For more details, see Supplementary Materials.

## Data Availability

The program can be accessed at at Zenodo, https://doi.org/10.5281/zenodo.5639551 (accessed on 2 November 2021).

## Supplementary Materials

The following are available online at www.mdpi.com/article/10.3390/biology10111147/s1.
