# Supplementary material for: A Study of the Coevolution of Digital Organisms with an Evolutionary Cellular Automaton
Source: Biology (Basel). 2021 Nov 7;10(11):1147. doi: 10.3390/biology10111147 (PMC8614957; doi:10.3390/biology10111147)
Supplement: Supplementary file 1 [file biology-10-01147-s001.zip › biology-1411890-supplementary.pdf]

Initial state	*Represents*	*Process*	*What happens*	*Why it happens*	*Reference*
1LCFY.json	Law of Constant Final Yield	Intraspecific competition: organisms of the same species compete for the scarce resources of each cell.	The amount of resources in each cell controls the growth rate of the species.	By increasing 100 resources in each of the 100 cells, the organisms that survive (carrying capacity) in each generation increase by 100 x 100 = 10.000	[43,44]
1Pred.json	Simple predation	Predator *Neodiprion sertifer* eats its prey (the resources)	Numerical answer: if resources increase, *N. sertifer* increases<br>Functional answer: if you increase both resources and Fitness direct *N. sertifer* increases	By increasing the resources in each patch, *N. sertifer* is adjusted by increasing its number.<br>*N. sertifer* is adjusted per patch. The load capacity does not run out if the distribution aggregated (100r)	[46]
1Amen.json	Amensalism (with cost)	*Eucalyptus* sp. competes with other plants for resources.	*Eucalyptus* sp. secretes a substance that prevents and hinders the development of other plants.	*Eucalyptus* sp. and other plants have the same Direct fitness, but when they interact, eucalyptus, at a cost to him (his Indirect fitness) makes other plants have less offspring. *Eucalyptus* prevails.	Deducted by the user
1Amen2.json	Amensalism	Two *Saccharomyces cerevisiae* yeast strains: a killer strain and a sensitive strain.	The killer prevails at equal fitness. Although the interaction can stay a long time if the punishments are few.	The killer reduces the fitness of the sensitive. If both species concur in a cell, most are grouped (high flexibility for the Killer) and have the same fitness as ungrouped, but if there is any non-clustered sensitive, it is punished. All groupings are undone (minimum flexibility for the group).	[69]
1Exclu.json	Competitive exclusion principle	Two species competing for the same limited resource cannot coexist at constant population values.	*Paramecium aurelia* and *Paramecium caudatum*. After a phase of neutrality *P. aurelia* systematically led to *P. caudatum* extinction.	*P. aurelia* has more fitness than *P. caudatum*, 7 to 4 offspring	[49]
1Intra.json	Intraspecific competition	Organisms of a species sp. compete with each other for the scarce resources of each cell	The number of organisms increases until the carrying capacity, from there only some organisms survive and have offspring.	By natural selection, only a few can survive because resources are limited	Deducted by the user
1Neu.json	Neutralism	Describes the relationship between two species that interact but do not affect each other.	Species SP1 and SP2 interact with each other, but neither benefit nor harm	Both species are related but nothing is given.	[50]
1Para.json	Brood parasites	The cuckoo (*Cuculus canorus*) deposits an egg in the reed warbler's nest (*Rocephalus arundinaceus*), and it breeds it as its own.	It is an extinct interaction because the cuckoo eliminates the eggs of the reed warbler. The reed warmer extinguishes and extinguishes the cuckoo.	The interaction is maintained if the cuckoo/reed warbler ratio is low and there is a great fragmentation of habitat because it gives time to regenerate the reed warblers.	[47,48]
1Comm.json	Commensalism (Phoresy)	One of the species obtains a benefit, the other neither benefits nor is harmed.	Sharksucker is grouped with Shark. The sharksucker has a benefit in transport and food (more offspring) while the shark has no benefit or loss for the interaction.	When both species are found, both form a group (maximum flexibility of shark), the created group consumes and reproduces before ungrouping (minimum group flexibility). In the grouping, sharksucker has more offspring than if it had not been grouped.	[51]
1Colab.json	Intraspecific social collaboration	Three species of bees compete in a habitat: An eusocial - *Apis mellifera* - that without associating with those of its same species cannot live; another that is associated but can live individually; and another that is never associated.	With a small population, they usually become extinct in this order: eusocial <collaborators <individual. If the population is large enough, the three species of bees are kept alive for many generations.	When they are odd numbers, the collaborators and the eusocials have an unpaired in each cell, losing offspring concerning the individual ones. This effect is diminished when the population is large and when there is little fragmentation of the habitat. In this case, the species compete in genetic drift and can be in balance for many generations (more than 500).	[71,72]
1Eu2.json	Multilevel selection vs kin selection	Two species of eusocial bees compete with solitary bees. One of the eusocials is associated following the kin selection theory, and the other is grouped following the multilevel selection theory.	If there is no eusociality or there is no more fitness with eusociability, the species are in equilibrium (1 and 2). Only when there are advantages in eusociality (one more descendant) do eusocials prevail (3,4 and 5). For the grouped species to prevail, they must all be grouped by setting the Phenotypic Flexibility to the maximum (6 and 7).	Cooperation in itself does not give advantages, only if it provides more fitness is selected. Those who do not group due to low phenotypic flexibility have fewer offspring and decrease their biotic potential in the grouping. In contrast, in the association, all associate and have the maximum offspring from the beginning.	[73]
1Symb.json	Symbiosis or obligate mutualism	In the islands of Japan, a stinkbug and six distinct bacterial lineages associated with insect populations interact. The bugs are associated with obligate microbial mutualists on an island, and on other islands, bacteria are parasitized. If the bacteria have a common ancestor and are functionally equal to the bug, it is suggested that such essential symbionts have originated from free-living ancestors.	On each island, one type of interaction is positively selected. One uncultivable symbiont is fixed in temperate populations, and the other uncultivable symbiont coexists with four culturable symbionts subtropical populations.	When the bacteria are free-living, the interaction works as in parasitism. When there is obligate mutualism, the bacteria can only live as an endosymbiont, and without associating, it dies. Df = If = 0.	[17]
1Deri.json	Genetic drift (in genes)	Two alleles (A and B) can be in homozygosis (AA and BB), or in heterozygosis (AB).	They start with the same frequency, but as the generations advance, they vary in frequency. If the number of alleles becomes small enough, one allele may disappear, the other one being fixed, without a single heterozygous locus.	For sampling errors, allele frequencies vary. If we decrease sufficiently the number of alleles, any of the alleles can disappear. For example, if we go from 200 loci to 10 loci, the allele A or allele B, is extinguished, making the genes homozygous.	[77,78]
1DeriSp.json	Ecological drift (in species)	Two species, A and B, compete in a habitat with 100 patches. They have equal fitness and equal frequency.	Frequencies A and B vary to happen generations. If the populations become small enough, any of them may disappear.	Sampling errors vary the frequencies of the populations.	[72]
1Hardy.json	Hardy–Weinberg principle	This principle states that allele and genotype frequencies in a population will remain constant from generation to generation in the absence of genetic drift, mate choice, assortative mating, natural selection, sexual selection, mutation, gene flow, meiotic drive, genetic hitchhiking, population bottleneck, founder effect and inbreeding.	The frequencies of alleles and genotypes are kept constant, for three alleles, until it reaches the limit of alleles that fit in the patch.	As in the previous case of genetic drift, the frequency of allele populations does not vary until natural selection appears.	[70]
1Pavo3.json and 1Pavo2.json	Kin selection (1)	In *Meleagris gallopavo*, pairs of males of the same age form coalitions to woo females and defend those females from other males. Only the dominant male has offspring. The males of these coalitions have a kinship coefficient of 0.42 (close to r of full siblings). Dominant males (in pairs) mate with more females and leave more offspring on average than solitary males (9 vs. 0.7 offspring).	We start from two male turkey lineages: those that form a dominant and subordinate male coalition and those that are solitary. On average, the coalition has more offspring (2, 3 offspring). The resources of each cell represent female turkeys, and each cell represents a nuptial display site or leks. The lineage of the solitaires is quickly extinguished.	The fitness of the turkey coalitions is higher and, by competition, excludes the lineage of the solitaries.	[81]
1Squi.json	Kin selection (2)	There were five adoptions among 2,230 litters over 19 years, in asocial red squirrels (*Tamiasciurus hudsonicus*). Adoptions were always between kin, while orphans without nearby kin were never adopted. It is unclear why an adopting parent should incur the costs of rearing extra young, if not by applying Hamilton's rule rB > C	We implement a model relative to the data collected in the research. The factor Q (implemented as a species that is not extinct and whose population is one organism in all generations) causes an additional organism (A) (adopted) to survive. In contrast, species B does not have this advantage.	Both species compete in 40 simulations for 10,000 years (2,631 generations). A survives B in 18 simulations, B to A in 13 simulations, and both remain in 9 simulations.	[82]
1Sex2.json and 1Sex3.json	The evolution of sex	One sexual species competes with another asexual. Male and female singles do not have offpring. If they associate they have 4 offpring, just like asexuals.	If there are no pathoges ("*NumberOfItems*" = 0) or the epidemic ends, asexuals prevail. If the pathogen is more lethal or more contagious among asexuals, sexual ones prevail.	Due to the Quorum decreasing effect (singles do not have offspring), asexuals always prevail, unless external factors, such as a pathogen or virus, act.	[87]
1Fish.json	Fisher's principle	The Fisher principle shows that the 1: 1 ratio sex is the evolutionarily stable strategy (ESS)	We simulate three sexual populations with the same fitness. Male and female singles do not have offspring. If they associate, they have six offspring, but in different ratios: 3:3, 2:4, and 4:2. According to Fisher's principle, the 3:3 ratio always prevails.	The bias between males and females is more significant when the sex ratio is not 1: 1, so the "quorum decreasing" effect is greater and more singles do not have offspring.	[93]
1Proto.json	Proto-Cooperation	Three predators (SP1, SP2 and SP3) compete for prey (the resources of the 100 cells). SP1 and SP2 can collaborate and get one more descendant, SP3 cannot.	Non-cooperating SP3 species becomes extinct	Proto-cooperation gives more fitness (one more descendant) ends up extinguishing the one that does not cooperate due to competitive exclusion	[52]

Law of Constant Final Yield

Intraspecific Competition

go back

Training dataset	[43,44]
Initial State Name	1LCFY.json
Terminal Command or Rank	rae4.py --outDir=1LCFY Paper1/1LCFY --setRandomSeed=1 --NumberOfRsrcsInEachCell=[100:1000:100]
Simulation data	Sheet 1LCFYdata

1LCFY.json — AE-4


```
{
    "NumberOfCells": 100,
    "NumberOfRsrcsInEachCell": 100,
    "MultilevelDeath1Percent": 0.00,
    "LambdaForEgoism": 0.0,
    "Distribution": "100n",
    "species": [
        {
        "id": "A",
        "NumberOfItems": 100,
        "DirectOffspring": 4,
        "Distribution": "100n",
        "GroupPartner": [],
        "PhenotypicFlexibility": 0.0,

        "AssociatedSpecies":   [],
        "IndirectOffspring": 0,
        "FitnessVariationLimit": 0
        }
    ]
}
```


Rank: From 100 to 1000

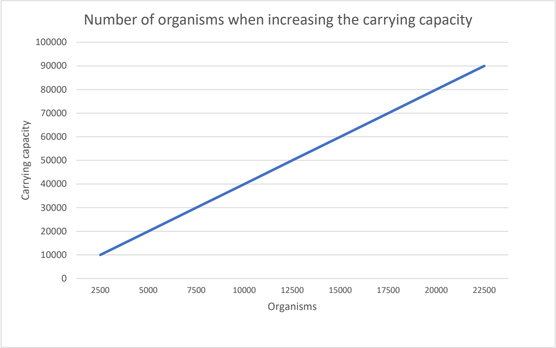

Predation

Functional and numerical responses with different distributions

go back

Training dataset	[46]
Initial State Name	1Pred.json
Terminal Command or Rank	ae4.py Paper1/1Pred
Simulation data	[46]

1

```
1Pred.json — AE-4
1Pred.json
{
    "NumberOfCells": 100,
    "NumberOfRsrcsInEachCell": 100,
    "Distribution": "100n",
    "species": [
        {
        "id": "N.sertifer",
        "NumberOfItems": 100,
        "DirectOffspring": 3,
        "GroupPartners": [],
        "PhenotypicFlexibility": 0.0,

        "AssociatedSpecies":   [],
        "IndirectOffspring": 0,
        "FitnessVariationLimit": 0
        }
    ]
}
```

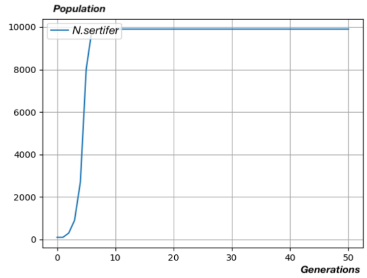

With 100 of resources for each cell and DirectOffspring = 3	
Terminal Command or Rank:	ae4.py Paper1/1Pred --numGen=50 -p

2

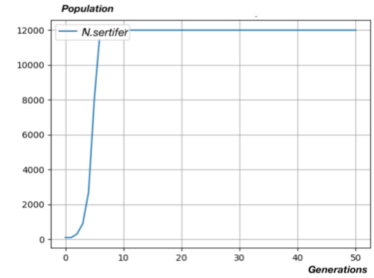

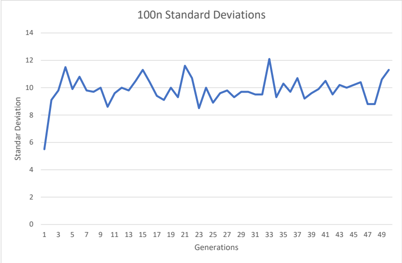

With 120 of resources for each cell and Direct = 3 NUMERICAL RESPONSE	
Terminal Command or Rank:	ae4.py Paper1/1Pred --numGen=50 -p --NumberOfRsrcsInEachCell=120

3

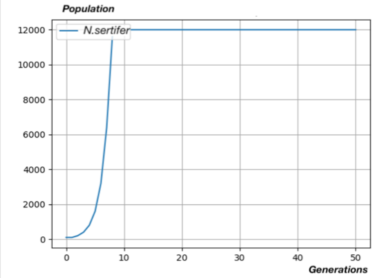

With 120 of resources for each cell and Direct = 2 NUMERICAL RESPONSE AND UNIFORM DISTRIBUTION	
Terminal Command or Rank:	ae4.py Paper1/1Pred --numGen=50 -p --NumberOfRsrcsInEachCell=120 --species="0;DirectOffspring=2"

4

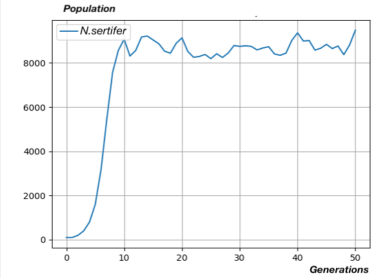

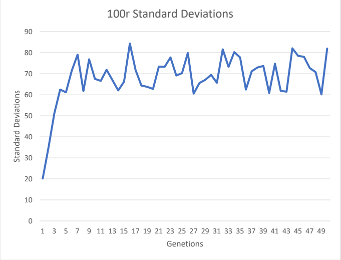

With 120 of resources for each cell and Direct = 3 AGGREGATED DISTRIBUTION	
Terminal Command or Rank:	ae4.py Paper1/1Pred --numGen=50 -p --NumberOfRsrcsInEachCell=120 --species="0;DirectOffspring=2" --Distribution=100r

Parasitism
Brood parasites

go back

Training dataset:	[47,48]
Initial State Name	1Para.json
Terminal Command or Rank:	ae4.py Paper1/1Para
Simulation data	

1

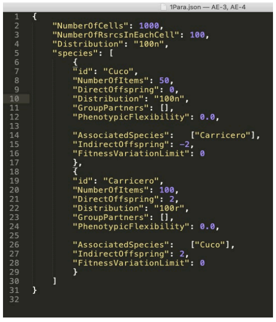
1Para.json — AE-3, AE-4

```
{
    "NumberOfCells": 1000,
    "NumberOfRsrcsInEachCell": 100,
    "Distribution": "100n",
    "species": [
        {
        "id": "Cuco",
        "NumberOfItems": 50,
        "DirectOffspring": 0,
        "Distribution": "100n",
        "GroupPartners": [],
        "PhenotypicFlexibility": 0.0,

        "AssociatedSpecies":   ["Carricero"],
        "IndirectOffspring": -2,
        "FitnessVariationLimit": 0
        },
        {
        "id": "Carricero",
        "NumberOfItems": 100,
        "DirectOffspring": 2,
        "Distribution": "100r",
        "GroupPartners": [],
        "PhenotypicFlexibility": 0.0,

        "AssociatedSpecies":   ["Cuco"],
        "IndirectOffspring": 2,
        "FitnessVariationLimit": 0
        }
    ]
}
```

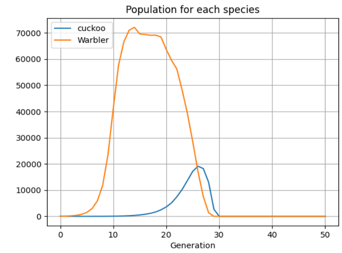

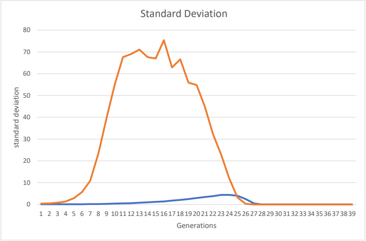

The cuckoo disperses more evenly distributed than the reed warbler (100n vs 100r) extinguishes it and the cuckoo extinguishes	
Terminal Command or Rank:	ae4.py Paper1/1Para -p --numGen=50 --setRandomSeed=0

2

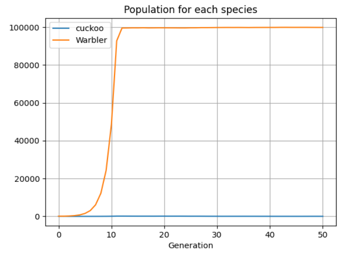

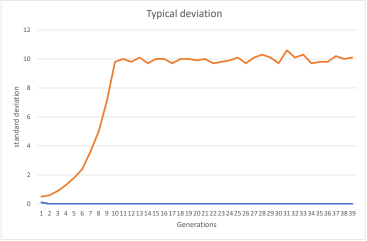

The cuckoo disperses less evenly distributed than the reed warbler (100r vs 100n) there is stable equilibrium	
Terminal Command or Rank:	ae4.py Paper1/1Para -p --numGen=50 --species="0;Distribution=100r;1;Distribution=100n"

3

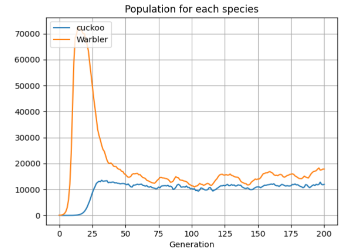

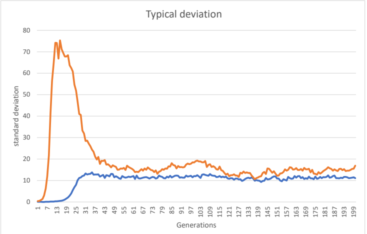

Both populations disperse equally, forming groups (100r): There is equilibrium	
Terminal Command or Rank:	ae4.py Paper1/1Para -p --numGen=200 --species="0;Distribution=100r;1;Distribution=100r" --setRandomSeed=2

4

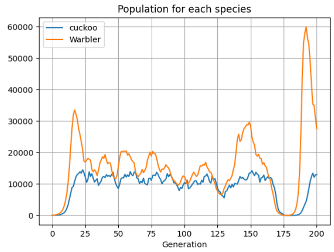

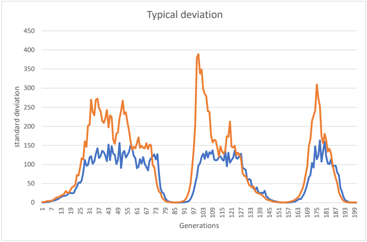

More fragmented habitat (from 1000 to 100 cells): there are cycles of near extinction of both populations	
Terminal Command or Rank:	ae4.py Paper1/1Para -p --numGen=200 --species="0;Distribution=100r;1;Distribution=100r" --setRandomSeed=2 --NumberOfCells=100 --NumberOfRsrcsInEachCell=1000

5

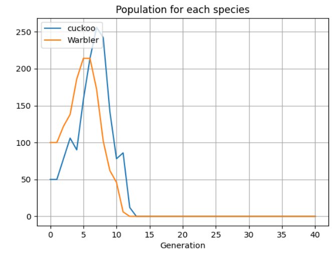

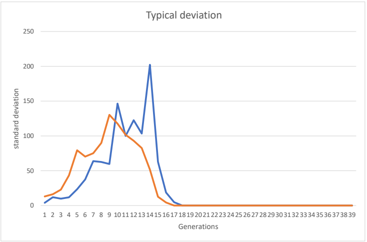

Even more fragmented habitat (1000 to 10 cells): both populations are rapidly becoming extinct.	
Terminal Command or Rank:	ae4.py Paper1/1Para -p --numGen=40 --species="0;Distribution=100r;1;Distribution=100r" --setRandomSeed=2 --NumberOfCells=10 --NumberOfRsrcsInEachCell=10000

Amensalism

Simple

go back

Training dataset	
Initial State Name	1Amen.json
Terminal Command or Rank	ae4.py Paper1/1Amen
Simulation data	

1Amen.json — AE-4

```
{
    "NumberOfCells": 100,
    "NumberOfRsrcsInEachCell": 100,
    "Distribution": "100n",
    "species": [
        {
        "id": "Eucalyptus",
        "NumberOfItems": 100,
        "DirectOffspring": 5,
        "Distribution": "100n",
        "GroupPartners": [],
        "PhenotypicFlexibility": 0.0,

        "AssociatedSpecies":   ["Other"],
        "IndirectOffspring": -2,
        "FitnessVariationLimit": 0
        },
        {
        "id": "Other",
        "NumberOfItems": 100,
        "DirectOffspring": 5,
        "Distribution": "100n",
        "GroupPartners": [],
        "PhenotypicFlexibility": 0.0,

        "AssociatedSpecies":   [],
        "IndirectOffspring": 0,
        "FitnessVariationLimit": 0
        }
    ]
}
```

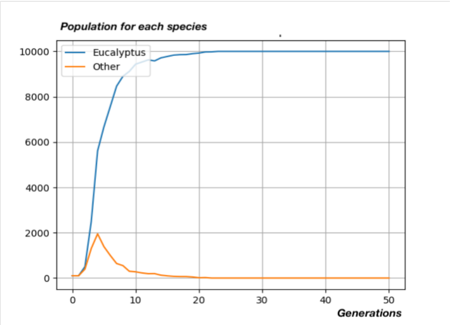

Eucalyptus and other plants	
Terminal Command or Rank:	ae4.py Paper1/1Amen --numGen=50 -p

Amensalism

With grouping

go back

Training dataset	69]
Initial State Name	1Amen2.json
Terminal Command or Rank	ae4.py Paper1/1Amen2
Simulation data	

1Amen2.json — AE-3, AE-4

```
{
    "NumberOfCells": 10,
    "NumberOfRsrcsInEachCell": 1000,
    "Distribution": "100n",
    "species": [
        {
        "id": "Killer",
        "NumberOfItems": 100,
        "DirectOffspring": 3,
        "Distribution": "100n",
        "GroupPartners": ["Sensitive"],
        "PhenotypicFlexibility": 0.10,

        "AssociatedSpecies":   [],
        "IndirectOffspring": 0,
        "FitnessVariationLimit": 0
        },
        {
        "id": "Sensitive",
        "NumberOfItems": 100,
        "DirectOffspring": 3,
        "Distribution": "100n",
        "GroupPartners": [],
        "PhenotypicFlexibility": 0.0,

        "AssociatedSpecies":   [],
        "IndirectOffspring": 0,
        "FitnessVariationLimit": 0
        },
        {
        "id": "Killer|Sensitive",
        "NumberOfItems": 0,
        "DirectOffspring": 3,
        "Distribution": "100n",
        "GroupPartners": [],
        "PhenotypicFlexibility": 0.0,

        "AssociatedSpecies":   ["Sensitive"],
        "IndirectOffspring": -2,
        "FitnessVariationLimit": 0
        }
    ]
```

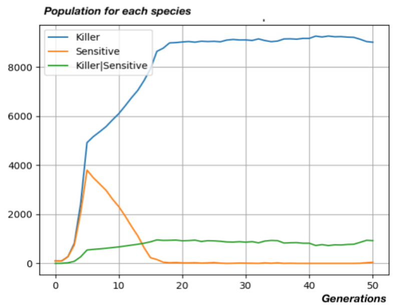

When the grouped killer kills the sensitive	
Terminal Command or Rank:	ae4.py Paper1/1Amen2 --numGen=50 -p

Competitive exclusion principle

go back

Training dataset	[47]
Initial State Name	1Exclu.json
Terminal Command or Rank	ae4.py Paper1/1Exclu --numGen=50 --verbose -p
Simulation data	

1Exclu.json


```json
{
    "NumberOfCells": 10,
    "NumberOfRsrcsInEachCell": 100,
    "Distribution": "100n",
    "species": [
        {
        "id": "P.aurelia",
        "NumberOfItems": 100,
        "DirectOffspring": 7,
        "Distribution": "100n",
        "GroupPartners": [],
        "PhenotypicFlexibility": 0.0,

        "AssociatedSpecies":   [],
        "IndirectOffspring": 0,
        "FitnessVariationLimit": 0
        },
        {
        "id": "P.caudatum",
        "NumberOfItems": 100,
        "DirectOffspring": 4,
        "Distribution": "100n",
        "GroupPartners": [],
        "PhenotypicFlexibility": 0.0,

        "AssociatedSpecies":   [],
        "IndirectOffspring": 0,
        "FitnessVariationLimit": 0
        }
    ]
}
```


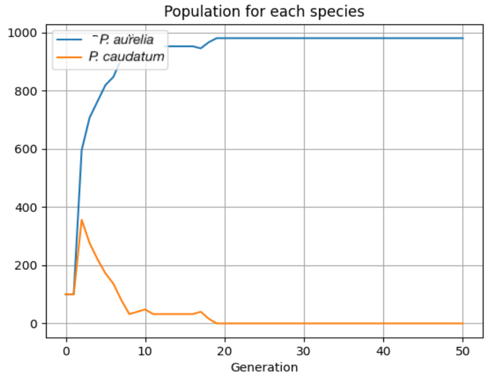

Intraspecific competition

Training dataset	
Initial State Name	1Intra.json
Terminal Command or Rank	ae4.py Paper1/1Intra -p
Simulation data	

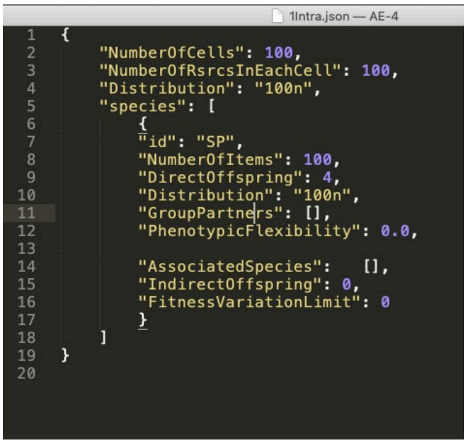


```
{
    "NumberOfCells": 100,
    "NumberOfRsrcsInEachCell": 100,
    "Distribution": "100n",
    "species": [
        {
        "id": "SP",
        "NumberOfItems": 100,
        "DirectOffspring": 4,
        "Distribution": "100n",
        "GroupPartners": [],
        "PhenotypicFlexibility": 0.0,

        "AssociatedSpecies":    [],
        "IndirectOffspring": 0,
        "FitnessVariationLimit": 0
        }
    ]
}
```

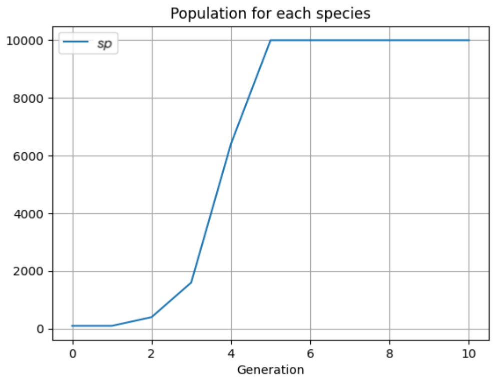

Neutralism

go back

Training dataset	[50]
Initial State Name	1Neu.json
Terminal Command or Rank	ae4.py Paper1/1Neu -p --numGen=100
Simulation data	

1Neu.json — AE-4


```json
{
    "NumberOfCells": 100,
    "NumberOfRsrcsInEachCell": 100,
    "Distribution": "100n",
    "species": [
        {
        "id": "SP1",
        "NumberOfItems": 100,
        "DirectOffspring": 2,
        "Distribution": "100n",
        "GroupPartners": [],
        "PhenotypicFlexibility": 0.0,

        "AssociatedSpecies":   ["SP2"],
        "IndirectOffspring": 0,
        "FitnessVariationLimit": 0
        },
        {
        "id": "SP2",
        "NumberOfItems": 100,
        "DirectOffspring": 2,
        "Distribution": "100n",
        "GroupPartners": [],
        "PhenotypicFlexibility": 0.0,

        "AssociatedSpecies":   ["SP1"],
        "IndirectOffspring": 0,
        "FitnessVariationLimit": 0
        }
    ]
}
```


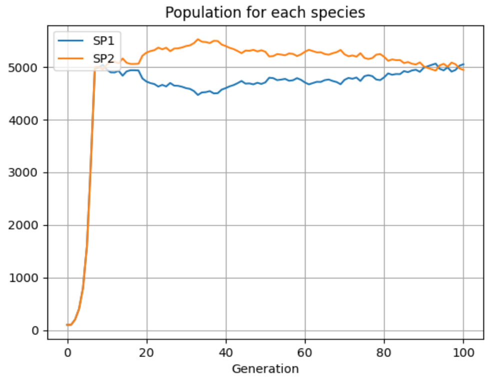

Commensalism

Sharksucker

go back

Training dataset	[51]
Initial State Name	1Comm.json
Terminal Command or Rank	ae4.py Paper1/1Comm
Simulation data	[51]

1Comm.json — AE-3, AE-4


```
{
    "NumberOfCells": 100,
    "NumberOfRsrcsInEachCell": 10000,
    "Distribution": "100n",
    "species": [
        {
        "id": "Sharksucker",
        "NumberOfItems": 100,
        "DirectOffspring": 3,

        "GroupPartners": ["Shark"],
        "PhenotypicFlexibility": 0.9,

        "AssociatedSpecies":   [],
        "IndirectOffspring": 0,
        "FitnessVariationLimit": 0
        },
        {
        "id": "Shark",
        "NumberOfItems": 100,
        "DirectOffspring": 70,

        "GroupPartners": [],
        "PhenotypicFlexibility": 0.0,

        "AssociatedSpecies":   [],
        "IndirectOffspring": 0,
        "FitnessVariationLimit": 0
        },
        {
        "id": "Sharksucker|Shark",
        "NumberOfItems": 0,
        "DirectOffspring": 70,

        "GroupPartners": [],
        "PhenotypicFlexibility": 0.0,

        "AssociatedSpecies":   [],
        "IndirectOffspring": 0,
        "FitnessVariationLimit": 0
```


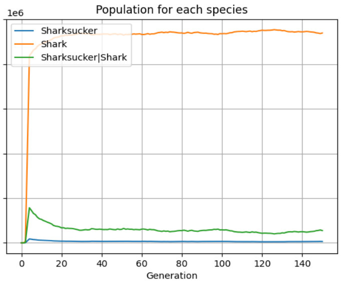

Uniform distribution 100n	
Terminal Command or Rank:	ae4.py Paper1/1Comm --numGen=150 -p

Theories on Eusociality
Multilevel selection vs kin selection

go back

Training dataset	[73]
Initial State Name	1Eu2.json
Terminal Command or Rank	ae4.py Paper1/1Eu2
Simulation data	

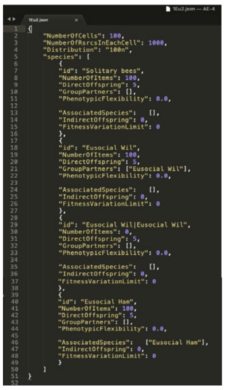


```
{
    "NumberOfCells": 100,
    "NumberOfRsrcsInEachCell": 1000,
    "Distribution": "100n",
    "species": [
        {
        "id": "Solitary bees",
        "NumberOfItems": 100,
        "DirectOffspring": 5,
        "GroupPartners": [],
        "PhenotypicFlexibility": 0.0,

        "AssociatedSpecies":   [],
        "IndirectOffspring": 0,
        "FitnessVariationLimit": 0
        },
        {
        "id": "Eusocial Wil",
        "NumberOfItems": 100,
        "DirectOffspring": 5,
        "GroupPartners": ["Eusocial Wil"],
        "PhenotypicFlexibility": 0.0,

        "AssociatedSpecies":   [],
        "IndirectOffspring": 0,
        "FitnessVariationLimit": 0
        },
        {
        "id": "Eusocial Wil|Eusocial Wil",
        "NumberOfItems": 0,
        "DirectOffspring": 5,
        "GroupPartners": [],
        "PhenotypicFlexibility": 0.0,

        "AssociatedSpecies":   [],
        "IndirectOffspring": 0,
        "FitnessVariationLimit": 0
        },
        {
        "id": "Eusocial Ham",
        "NumberOfItems": 100,
        "DirectOffspring": 5,
        "GroupPartners": [],
        "PhenotypicFlexibility": 0.0,

        "AssociatedSpecies":   ["Eusocial Ham"],
        "IndirectOffspring": 0,
        "FitnessVariationLimit": 0
        }
    ]
}
```


1

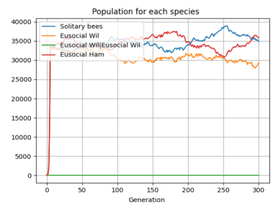

Without eusociality there is balance

Terminal Command or Rank:	ae4.py Paper1/1Eu2 --numGen=300 -p

2

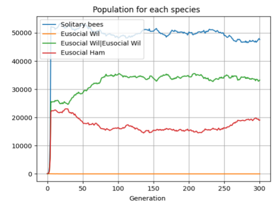

Without advantages in eusociability, there is balance

Terminal Command or Rank:	ae4.py Paper1/1Eu2 --numGen=300 -p --species="1;PhenotypicFlexibility=1;2;PhenotypicFlexibility=1;3;DirectOffspring=0;IndirectOffspring=5"

3

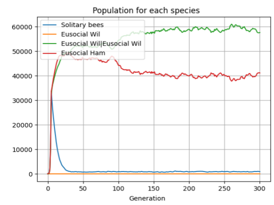

Both species of eusocial bees prevail for the same

Terminal Command or Rank:	ae4.py Paper1/1Eu2 --numGen=300 -p --species="1;PhenotypicFlexibility=1;2;DirectOffspring=6;3;DirectOffspring=0;IndirectOffspring=6"

4

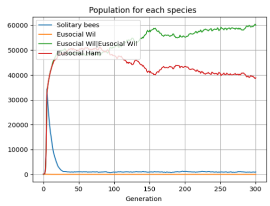

Both species of eusocial bees prevail for the same

Terminal Command or Rank:	ae4.py Paper1/1Eu2 --numGen=300 -p --species="1;PhenotypicFlexibility=1;2;DirectOffspring=6;PhenotypicFlexibility=1;3;DirectOffspring=0;IndirectOffspring=6"

5

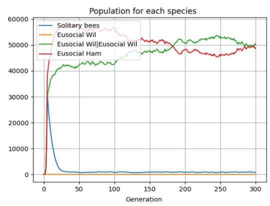

Both species of eusocial bees prevail for the same

Terminal Command or Rank:	ae4.py Paper1/1Eu2 --numGen=300 -p --species="1;PhenotypicFlexibility=1;2;DirectOffspring=6;PhenotypicFlexibility=0.5;3;DirectOffspring=1;IndirectOffspring=5"00

6

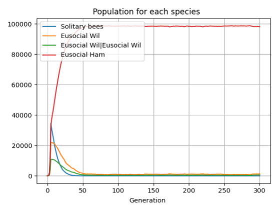

The bee species that follows the kin selection prevails

Terminal Command or Rank:	ae4.py Paper1/1Eu2 --numGen=300 -p --species="1;PhenotypicFlexibility=0.5;2;DirectOffspring=6;3;DirectOffspring=0;IndirectOffspring=6"

7

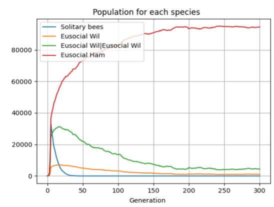

The bee species that follows the kin selection prevails

Terminal Command or Rank:	ae4.py Paper1/1Eu2 --numGen=300 -p --species="1;PhenotypicFlexibility=0.9;2;DirectOffspring=6;PhenotypicFlexibility=0;3;DirectOffspring=0;IndirectOffspring=6"

Intraspecific social collaboration
Non-collaboration *vs.* Association *vs.* Eusociality

go back

Training dataset	[71,72]
Initial State Name	1Colab.json
Terminal Command or Rank	ae4.py Paper1/1Colab
Simulation data	

1

1Colab.json — AE-4


```json
{
    "NumberOfCells": 100,
    "NumberOfRsrcsInEachCell": 1000,
    "Distribution": "100r",
    "species": [
        {
        "id": "Solitary bees",
        "NumberOfItems": 100,
        "DirectOffspring": 5,
        "GroupPartners": [],
        "PhenotypicFlexibility": 0.0,

        "AssociatedSpecies":   [],
        "IndirectOffspring": 0,
        "FitnessVariationLimit": 0
        },
        {
        "id": "Eusocial bess",
        "NumberOfItems": 100,
        "DirectOffspring": 0,
        "GroupPartners": [],
        "PhenotypicFlexibility": 0.0,

        "AssociatedSpecies":   ["Eusocial bess"],
        "IndirectOffspring": 5,
        "FitnessVariationLimit": 0
        },
        {
        "id": "Collaborating bees",
        "NumberOfItems": 100,
        "DirectOffspring": 3,
        "GroupPartners": [],
        "PhenotypicFlexibility": 0.0,

        "AssociatedSpecies":   ["Collaborating bees"],
        "IndirectOffspring": 2,
        "FitnessVariationLimit": 0
        }
    ]
}
```


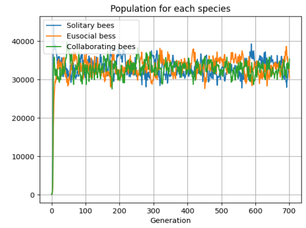

Aggregated distribution, fragmented habitat	
Terminal Command or Rank:	ae4.py Paper1/1Colab -p --numGen=700 --Distribution=100r

2

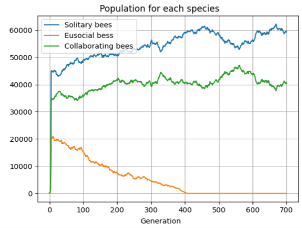

Uniform distribution, fragmented habitat	
Terminal Command or Rank:	ae4.py Paper1/1Colab -p --numGen=700 --Distribution=100n

3

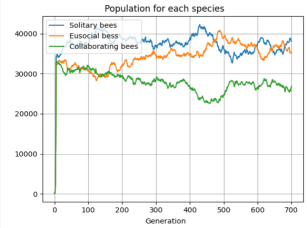

Uniform distribution, habitat little fragmented	
Terminal Command or Rank:	ae4.py Paper1/1Colab -p --numGen=700 --Distribution=100n --NumberOfCells=5 --NumberOfRsrcsInEachCell=20000

4

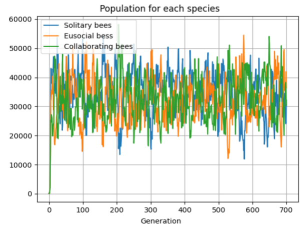

Aggregated distribution, little fragmented habitat	
Terminal Command or Rank:	ae4.py Paper1/1Colab -p --numGen=700 --Distribution=100r --NumberOfCells=5 --NumberOfRsrcsInEachCell=20000

5

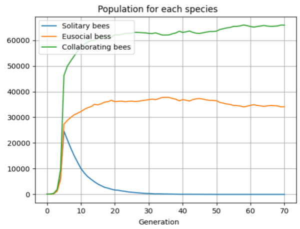

Solitary less efficient, more uniform distribution	
Terminal Command or Rank:	ae4.py Paper1/1Colab -p --numGen=70 --Distribution=100n --species="0;DirectOffspring=4"

6

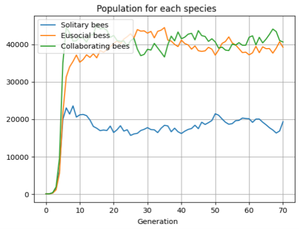

Solitary less efficient, less uniform distribution	
Terminal Command or Rank:	ae4.py Paper1/1Colab -p --numGen=70 --Distribution=100r --species="0;DirectOffspring=4"

Interspecific social collaboration

go back

Training dataset	[17]
Initial State Name	1Symb.json
Terminal Command or Rank	ae4.py Paper1/1Symb
Simulation data	[17]

1

1Symb.json — AE-3, AE-4


```
{
    "NumberOfCells": 100,
    "NumberOfRsrcsInEachCell": 1000,
    "Distribution": "100n",
    "species": [
        {
        "id": "Environmental bacteria",
        "NumberOfItems": 100,
        "DirectOffspring": 3,

        "GroupPartners": [],
        "PhenotypicFlexibility": 0.0,

        "AssociatedSpecies":   ["Stinkbug"],
        "IndirectOffspring": 3,
        "FitnessVariationLimit": 0
        },
        {
        "id": "Stinkbug",
        "NumberOfItems": 100,
        "DirectOffspring": 0,

        "GroupPartners": ["Environmental bacteria"],
        "PhenotypicFlexibility": 0.5,

        "AssociatedSpecies":   [],
        "IndirectOffspring": 0,
        "FitnessVariationLimit": 0
        },
        {
        "id": "Stinkbug|Environmental bacteria",
        "NumberOfItems": 0,
        "DirectOffspring": 3,

        "GroupPartners": [],
        "PhenotypicFlexibility": 0.00,

        "AssociatedSpecies":   [],
        "IndirectOffspring": 0,
        "FitnessVariationLimit": 0
        }
```


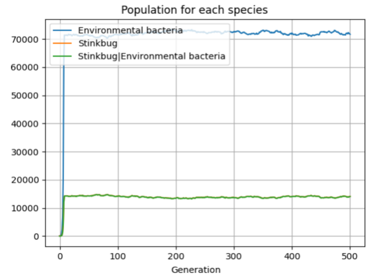

There is no symbiosis: PhenotypicFlexibility of the group = 0. Bedbug PhenotypicFlexibility = 0.5	
Terminal Command or Rank:	ae4.py Paper1/1Symb -p --numGen=500

2

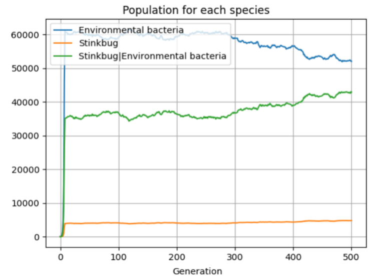

There is no symbiosis: PhenotypicFlexibility of the group = 0. Bedbug PhenotypicFlexibility = 0.9	
Terminal Command or Rank:	ae4.py Paper1/1Symb -p --numGen=500 --species="1;PhenotypicFlexibility=0.9"

3

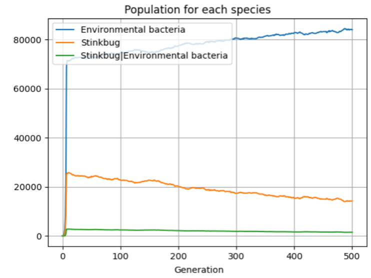

There is no symbiosis: PhenotypicFlexibility of the group = 0. Bedbug PhenotypicFlexibility = 0.1	
Terminal Command or Rank:	ae4.py Paper1/1Symb -p --numGen=500 --species="1;PhenotypicFlexibility=0.1"

4

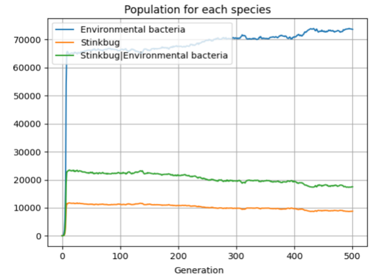

Symbiosis at 50%: PhenotypicFlexibility of the group = 0.5. Bedbug PhenotypicFlexibility = 0.5	
Terminal Command or Rank:	ae4.py Paper1/1Symb -p --numGen=500 --species="2;PhenotypicFlexibility=0.5"

5

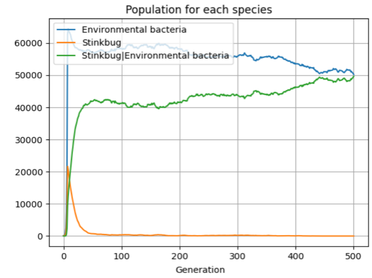

Symbiosis only: PhenotypicFlexibility of the group = 1. Bedbug PhenotypicFlexibility = 0.1	
Terminal Command or Rank:	ae4.py Paper1/1Symb -p --numGen=500 --species="1;PhenotypicFlexibility=0.1;2;PhenotypicFlexibility=1"

Random Genetic drift

go back

Training dataset	[77,78]
Initial State Name	1Deri.json
Terminal Command or Rank	ae4.py Paper1/1Deri
Simulation data	`77,78

1

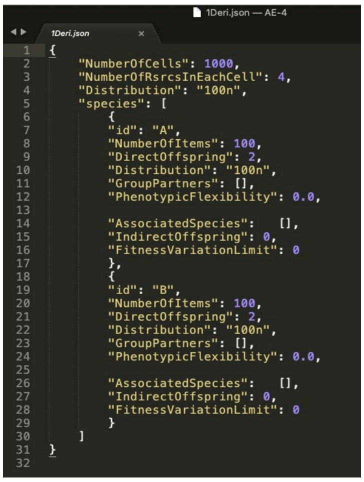


```
{
    "NumberOfCells": 1000,
    "NumberOfRsrcsInEachCell": 4,
    "Distribution": "100n",
    "species": [
        {
        "id": "A",
        "NumberOfItems": 100,
        "DirectOffspring": 2,
        "Distribution": "100n",
        "GroupPartners": [],
        "PhenotypicFlexibility": 0.0,

        "AssociatedSpecies":   [],
        "IndirectOffspring": 0,
        "FitnessVariationLimit": 0
        },
        {
        "id": "B",
        "NumberOfItems": 100,
        "DirectOffspring": 2,
        "Distribution": "100n",
        "GroupPartners": [],
        "PhenotypicFlexibility": 0.0,

        "AssociatedSpecies":   [],
        "IndirectOffspring": 0,
        "FitnessVariationLimit": 0
        }
    ]
}
```

2

For 1000 genes. For a large enough population (heterozygous)	
Terminal Command or Rank:	ae4.py Paper1/1Deri --numGen=600 -p

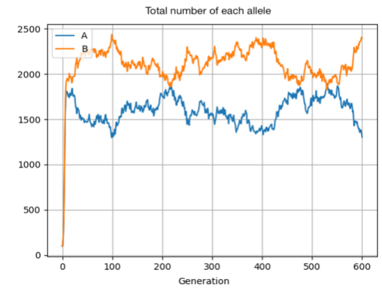

For 100 genes. For a small population (tends to be homozygous)	
Terminal Command or Rank:	ae4.py Paper1/1Deri --numGen=600 -p --NumberOfCells=100

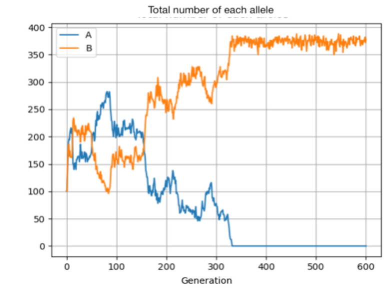

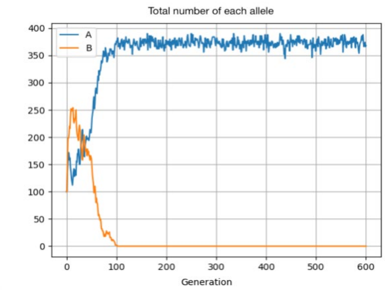

Ecological drift

In species

go back

Training dataset	[79]
Initial State Name	1DeriSP.json
Terminal Command or Rank	ae4.py Paper1/1DeriSP
Simulation data	

1DeriSP.json — AE-4

1DeriSP.json


```json
{
    "NumberOfCells": 100,
    "NumberOfRsrcsInEachCell": 20,
    "Distribution": "100n",
    "species": [
        {
        "id": "sp A",
        "NumberOfItems": 100,
        "DirectOffspring": 3,
        "Distribution": "100n",
        "GroupPartners": [],
        "PhenotypicFlexibility": 0.0,

        "AssociatedSpecies":   [],
        "IndirectOffspring": 0,
        "FitnessVariationLimit": 0
        },
        {
        "id": "sp B",
        "NumberOfItems": 100,
        "DirectOffspring": 3,
        "Distribution": "100n",
        "GroupPartners": [],
        "PhenotypicFlexibility": 0.0,

        "AssociatedSpecies":   [],
        "IndirectOffspring": 0,
        "FitnessVariationLimit": 0
        }
    ]
}
```


For 1800 organisms EEE	
Terminal Command or Rank:	ae4.py Paper1/1DeriSP --numGen=600 -p

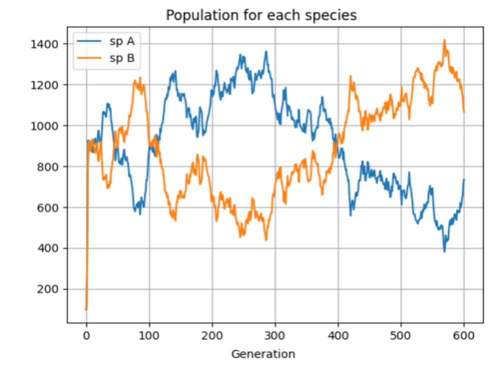

For 1800 organisms >SP BLUE	
Terminal Command or Rank:	ae4.py Paper1/1DeriSP --numGen=600 -p

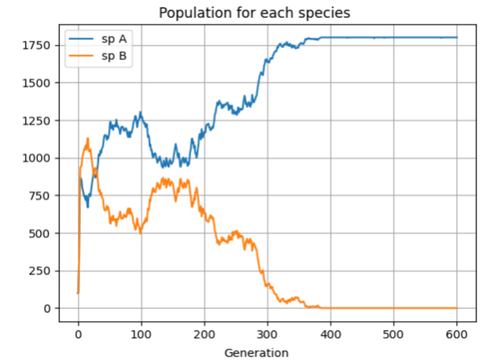

For 1800 organisms >SP ORANGE	
Terminal Command or Rank:	ae4.py Paper1/1DeriSP --numGen=600 -p

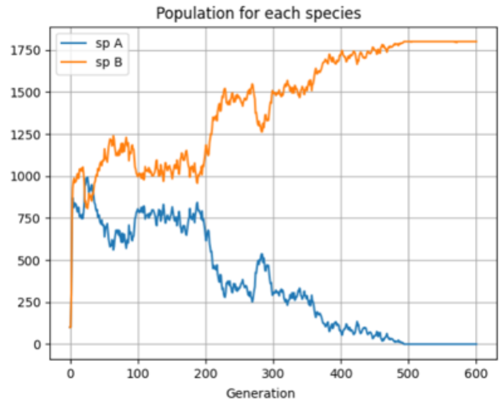

Hardy–Weinberg principle

go back

Training dataset	[70
Initial State Name	1Hardy.json
Terminal Command or Rank	rae4.py --outDir=Paper1/1HARDY Paper1/1Hardy --numGen=30 --NumberOfRsrcsInEachCell=[1000000:10000000:1000000]
Simulation data	

1Hardy.json — AE-3, AE-4


```
{
    "NumberOfCells": 1,
    "NumberOfRsrcsInEachCell": 1000000,
    "Distribution": "100n",
    "species": [
        {
        "id": "A",
        "NumberOfItems": 100,
        "DirectOffspring": 2,
        "Distribution": "100n",
        "GroupPartners": [],
        "PhenotypicFlexibility": 0.0,

        "AssociatedSpecies":   [],
        "IndirectOffspring": 0,
        "FitnessVariationLimit": 0
        },
        {
        "id": "a",
        "NumberOfItems": 100,
        "DirectOffspring": 2,
        "Distribution": "100n",
        "GroupPartners": [],
        "PhenotypicFlexibility": 0.0,

        "AssociatedSpecies":   [],
        "IndirectOffspring": 0,
        "FitnessVariationLimit": 0
        },
        {
        "id": "B",
        "NumberOfItems": 100,
        "DirectOffspring": 2,
        "Distribution": "100n",
        "GroupPartners": [],
        "PhenotypicFlexibility": 0.0,

        "AssociatedSpecies":   [],
        "IndirectOffspring": 0,
        "FitnessVariationLimit": 0
        }
    ]
```


Rank: From 100000 to 10000000

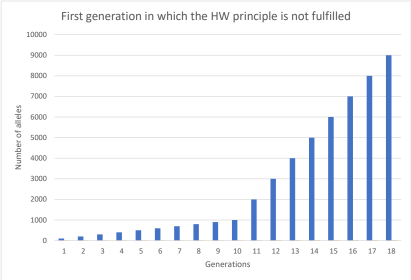

While the alleles that prevail because there are loci increase, the generation in which the HW principle is not fulfilled is delayed.	
Terminal Command or Rank:	rae4.py --outDir=Paper1/1HARDY Paper1/1Hardy --numGen=30 --NumberOfRsrcsInEachCell=[1000000:10000000:1000000]

Kin Selection
Cooperative courtship in wild turkeys

Training dataset	[81
Initial State Name	1Pavo3.json and 1Pavo2.json
Terminal Command or Rank	
Simulation data	[81

1

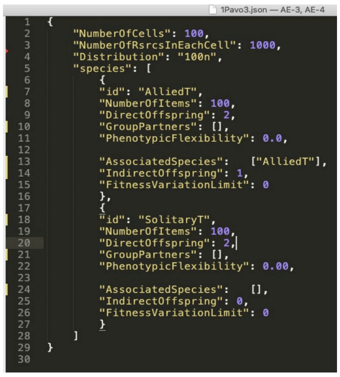

```
1Pavo3.json — AE-3, AE-4
{
    "NumberOfCells": 100,
    "NumberOfRsrcsInEachCell": 1000,
    "Distribution": "100n",
    "species": [
        {
        "id": "AlliedT",
        "NumberOfItems": 100,
        "DirectOffspring": 2,
        "GroupPartners": [],
        "PhenotypicFlexibility": 0.0,

        "AssociatedSpecies":    ["AlliedT"],
        "IndirectOffspring": 1,
        "FitnessVariationLimit": 0
        },
        {
        "id": "SolitaryT",
        "NumberOfItems": 100,
        "DirectOffspring": 2,
        "GroupPartners": [],
        "PhenotypicFlexibility": 0.00,

        "AssociatedSpecies":    [],
        "IndirectOffspring": 0,
        "FitnessVariationLimit": 0
        }
    ]
}
```

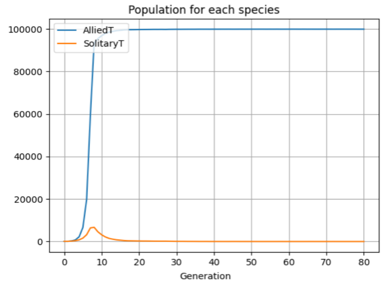

Allied turkeys only cooperate with themselves	
Terminal Command or Rank:	ae4.py Paper1/1Pavo3 --numGen=80 -p

2

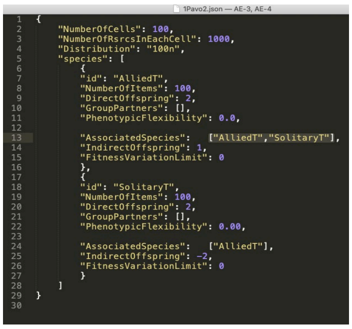

```
1Pavo2.json — AE-3, AE-4
{
    "NumberOfCells": 100,
    "NumberOfRsrcsInEachCell": 1000,
    "Distribution": "100n",
    "species": [
        {
        "id": "AlliedT",
        "NumberOfItems": 100,
        "DirectOffspring": 2,
        "GroupPartners": [],
        "PhenotypicFlexibility": 0.0,

        "AssociatedSpecies":    ["AlliedT","SolitaryT"],
        "IndirectOffspring": 1,
        "FitnessVariationLimit": 0
        },
        {
        "id": "SolitaryT",
        "NumberOfItems": 100,
        "DirectOffspring": 2,
        "GroupPartners": [],
        "PhenotypicFlexibility": 0.00,

        "AssociatedSpecies":    ["AlliedT"],
        "IndirectOffspring": -2,
        "FitnessVariationLimit": 0
        }
    ]
}
```

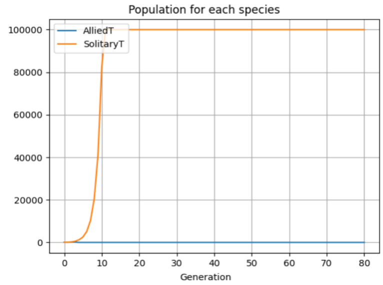

Allied turkeys cooperate with anyone	
Terminal Command or Rank:	ae4.py Paper1/1Pavo2 --numGen=80 -p

Kin Selection

Adopting kin enhances inclusive fitness in asocial red squirrels

go back

Training dataset	[82
Initial State Name	1Squi.json
Terminal Command or Rank	ae4.py Paper1/1Squi --numGen=2631 --saveExcel --verbose
Simulation data	[82

1Squi.json — AE-3, AE-4


```json
{
    "NumberOfCells": 446,
    "NumberOfRsrcsInEachCell": 6,
    "Distribution": "3n",
    "species": [
        {
        "id": "A",
        "NumberOfItems": 10,
        "DirectOffspring": 4,
        "GroupPartners": [],
        "PhenotypicFlexibility": 0.0,

        "AssociatedSpecies":   [],
        "IndirectOffspring": 0,
        "FitnessVariationLimit": 0
        },
        {
        "id": "B",
        "NumberOfItems": 10,
        "DirectOffspring": 4,
        "GroupPartners": [],
        "PhenotypicFlexibility": 0.0,

        "AssociatedSpecies":   [],
        "IndirectOffspring": 0,
        "FitnessVariationLimit": 0
        },
        {
        "id": "Q",
        "NumberOfItems": 1,
        "DirectOffspring": 1,
        "GroupPartners": [],
        "PhenotypicFlexibility": 0.0,

        "AssociatedSpecies":   ["A"],
        "IndirectOffspring": 1,
        "FitnessVariationLimit": 0
        }
    ]
}
```


Simulation data	***Equivalence***
Litter of 4	DirectOffpring=4
In 2,230 litters 5 adoptions are observed	The "Q" factor appears every 446 cells
One territory	One cell
Dispersion: 3 diameters of territory (2D)	Thirteen cells (1D): Distribution = 3n
10000 years	2631 generations

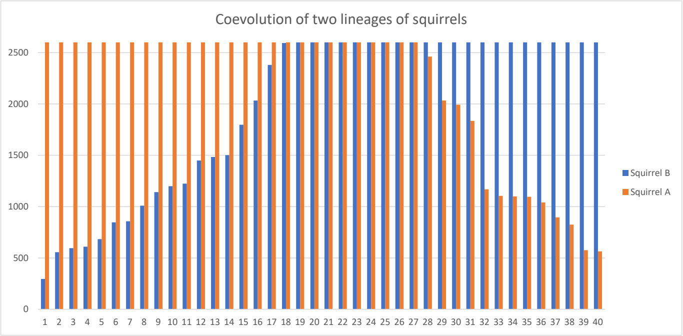

Sex
The evolution of sex

go back

Training dataset	[87]
Initial State Name	1Sex2.json and 1Sex3.json
Terminal Command or Rank	ae4.py Paper1/1Sex2 and ae4.py Paper1/1Sex3
Simulation data	[87]

1

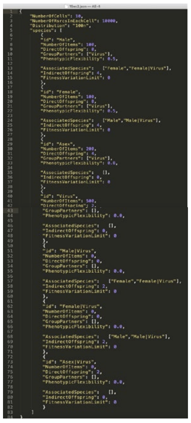

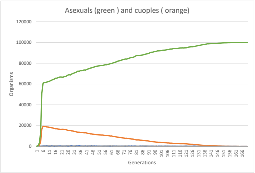

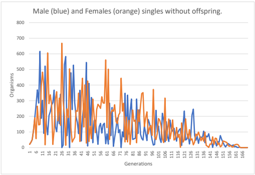

There are no pathogens: asexuals prevail	
Terminal Command or Rank:	ae4.py Paper1/1Sex2 --verbose --setRandomSeed=0 --numGen=170 --species="3;NumberOfItems=0" --saveExcel

2

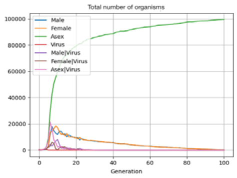

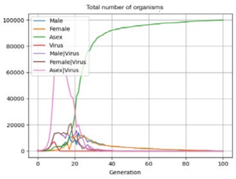

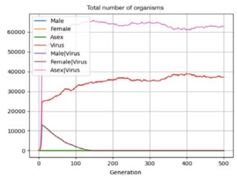

The pathogen infects everyone equally: asexuals prevail	
Terminal Command or Rank (left):	ae4.py Paper1/1Sex2 --verbose --setRandomSeed=0 --numGen=100 -p --species="2;PhenotypicFlexibility=0.5
Terminal Command or Rank (center):	ae4.py Paper1/1Sex2 --verbose --setRandomSeed=0 --numGen=100 -p --species="0;PhenotypicFlexibility=0.95;1;PhenotypicFlexibility=0.95;2;PhenotypicFlexibility=0.95"
Terminal Command or Rank (right):	ae4.py Paper1/1Sex2 --verbose --setRandomSeed=0 --numGen=500 -p --species="0;PhenotypicFlexibility=1;1;PhenotypicFlexibility=1;2;PhenotypicFlexibility=1"

3

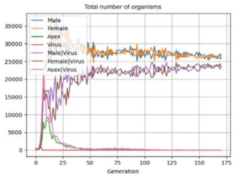

The pathogen infects the axesual more (0.8/0.5): the sexuals prevail	
Terminal Command or Rank:	ae4.py Paper1/1Sex2 --verbose --setRandomSeed=0 --numGen=170 -p

4

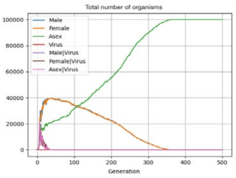

The pathogen infects the axesual more (0.75 vs 0.5): the asexuals prevail	
Terminal Command or Rank:	ae4.py Paper1/1Sex2 --verbose --setRandomSeed=0 --numGen=500 -p --species="2;PhenotypicFlexibility=0.75"

5

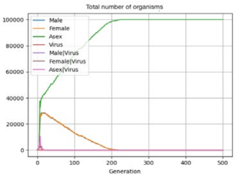

The pathogen infects everyone equally, but it is more lethal in asexuals (1 vs 2 DirectOffspring): asexuals prevail	
Terminal Command or Rank:	ae4.py Paper1/1Sex2 --verbose --setRandomSeed=0 --numGen=500 -p --species="2;PhenotypicFlexibility=0.5;6;DirectOffspring=1"

6

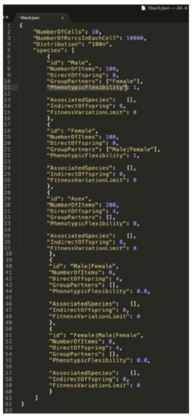

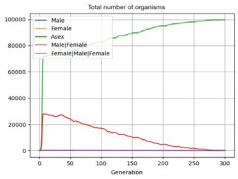

Asexuals prevail over sexuals even in polygamy	
Terminal Command or Rank:	ae4.py Paper1/1Sex3 -p --numGen=300

7

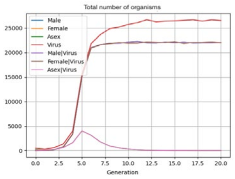

The pathogen infects everyone equally, but it Is much more lethal in asexuals (1 vs 3 DirectOffspring): sexuals prevail	
Terminal Command or Rank:	ae4.py Paper1/1Sex2 --verbose --setRandomSeed=0 --numGen=20 -p --species="2;PhenotypicFlexibility=0.5;4;DirectOffspring=3;5;DirectOffspring=3;6;DirectOffspring=1"

8

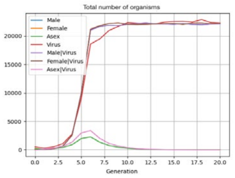

The pathogen infects a little more (0.5 vs 0.6) and is a little more lethal in asexuals (1 vs 2 DirectOffspring): sexuals prevail	
Terminal Command or Rank:	ae4.py Paper1/1Sex2 --verbose --setRandomSeed=0 --numGen=20 -p --species="2;PhenotypicFlexibility=0.6;4;DirectOffspring=2;5;DirectOffspring=2;6;DirectOffspring=1"

Fisher's principle

The 1:1 ratio is the evolutionarily stable strategy (ESS)

go back

Training dataset	[93]
Initial State Name	1Fish.json
Terminal Command or Rank	ae4.py Paper1/1Fish --numGen=30 -p --saveExcel
Simulation data	

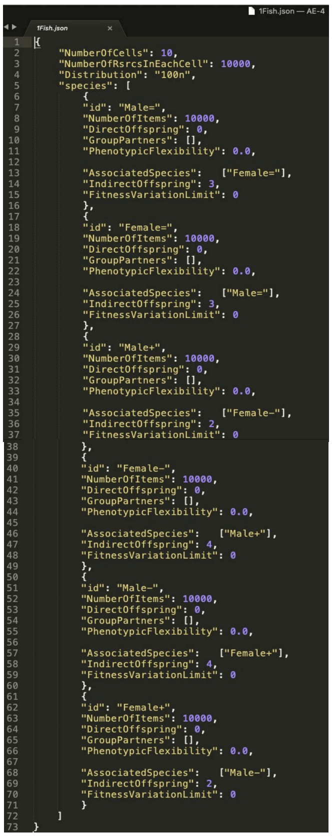
1Fish.json — AE-4

1Fish.json


```json
{
    "NumberOfCells": 10,
    "NumberOfRsrcsInEachCell": 10000,
    "Distribution": "100n",
    "species": [
        {
        "id": "Male=",
        "NumberOfItems": 10000,
        "DirectOffspring": 0,
        "GroupPartners": [],
        "PhenotypicFlexibility": 0.0,

        "AssociatedSpecies":    ["Female="],
        "IndirectOffspring": 3,
        "FitnessVariationLimit": 0
        },
        {
        "id": "Female=",
        "NumberOfItems": 10000,
        "DirectOffspring": 0,
        "GroupPartners": [],
        "PhenotypicFlexibility": 0.0,

        "AssociatedSpecies":    ["Male="],
        "IndirectOffspring": 3,
        "FitnessVariationLimit": 0
        },
        {
        "id": "Male+",
        "NumberOfItems": 10000,
        "DirectOffspring": 0,
        "GroupPartners": [],
        "PhenotypicFlexibility": 0.0,

        "AssociatedSpecies":    ["Female-"],
        "IndirectOffspring": 2,
        "FitnessVariationLimit": 0
        },
        {
        "id": "Female-",
        "NumberOfItems": 10000,
        "DirectOffspring": 0,
        "GroupPartners": [],
        "PhenotypicFlexibility": 0.0,

        "AssociatedSpecies":    ["Male+"],
        "IndirectOffspring": 4,
        "FitnessVariationLimit": 0
        },
        {
        "id": "Male-",
        "NumberOfItems": 10000,
        "DirectOffspring": 0,
        "GroupPartners": [],
        "PhenotypicFlexibility": 0.0,

        "AssociatedSpecies":    ["Female+"],
        "IndirectOffspring": 4,
        "FitnessVariationLimit": 0
        },
        {
        "id": "Female+",
        "NumberOfItems": 10000,
        "DirectOffspring": 0,
        "GroupPartners": [],
        "PhenotypicFlexibility": 0.0,

        "AssociatedSpecies":    ["Male-"],
        "IndirectOffspring": 2,
        "FitnessVariationLimit": 0
        }
    ]
}
```


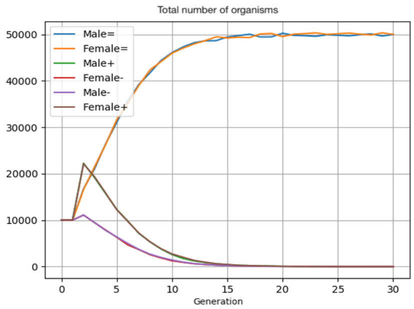

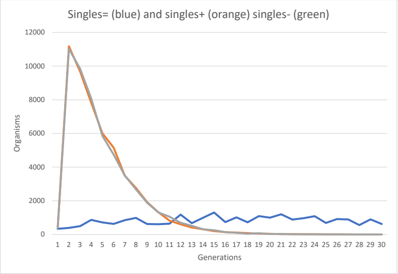

Proto-Cooperation

go back

Training dataset	[52
Initial State Name	1Proto.json
Terminal Command or Rank	ae4.py Paper1/1Proto
Simulation data	

1

1Proto.json — AE-4

1Proto.json

```
{
    "NumberOfCells": 100,
    "NumberOfRsrcsInEachCell": 100,
    "Distribution": "100n",
    "species": [
        {
        "id": "SP1",
        "NumberOfItems": 100,
        "DirectOffspring": 3,
        "GroupPartners": [],
        "PhenotypicFlexibility": 0.0,

        "AssociatedSpecies":   ["SP2"],
        "IndirectOffspring": 1,
        "FitnessVariationLimit": 0
        },
        {
        "id": "SP2",
        "NumberOfItems": 100,
        "DirectOffspring": 3,
        "GroupPartners": [],
        "PhenotypicFlexibility": 0.0,

        "AssociatedSpecies":   ["SP1"],
        "IndirectOffspring": 1,
        "FitnessVariationLimit": 0
        },
        {
        "id": "SP3",
        "NumberOfItems": 100,
        "DirectOffspring": 3,
        "GroupPartners": [],
        "PhenotypicFlexibility": 0.0,

        "AssociatedSpecies":   [],
        "IndirectOffspring": 0,
        "FitnessVariationLimit": 0
        }
```

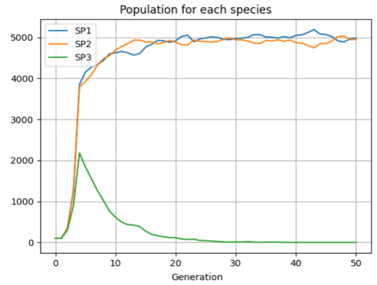

Species SP1 and SP2 obtain one more offspring when they collaborate, they prevail over SP3	
Terminal Command or Rank:	ae4.py Paper1/1Proto -p --numGen=50
